# Supplementary material for: Communication to adult patients undergoing cancer care by non-specialist nurses: a scoping review protocol
Source: BMJ Open. 2024 Mar 20;14(3):e081326. doi: 10.1136/bmjopen-2023-081326 (PMC10961544; doi:10.1136/bmjopen-2023-081326)
Supplement: Supplementary data [file bmjopen-2023-081326supp001.pdf]

Appendix 1 Scoping search results

On 24 October 2022, a scoping search was conducted to check if this scoping review (or any review) is in process or has been conducted in the past. JBI systematic review register, Prospero, Cochrane register, and PubMed was searched using the simple search term "cancer communication". The table below summarises the findings:

Database	Summary
Prospero	The search retrieved nine records.<br>No registered review of the proposed topic.
JBI systematic review register	Only three articles were retrieved. No article included cancer communication.
PubMed	After filtering by meta-analysis, reviews, and systematic reviews, the search term "cancer communication*" yielded 136 studies. No review was similar to this scoping review objectives.
Cochrane	The search retrieved 35 records. No review was similar to this scoping review objectives.

Appendix 2 Initial data extraction

MEDLINE

1	Describing the population of this study	Results
Participants	(MH "Nursing Staff+") OR (MH "Nurses+") OR (MH "Nursing+") OR (MH "Nursing Care+") OR (MH "Nurse Practitioners+") OR (MH "Nursing Staff, Hospital+") OR "nursing staff" OR nurs* OR "nursing care" OR "nurse practitioner*" OR "hospital nurse*" OR "non-speciali#ed nurse*"	633,365
2	Describing the concepts –Cancers	
Cancers	(MH "Neoplasms+") OR neoplasm* OR neoplasia Or tum#r* OR "Malignant neoplasm*" OR Malignan* OR cancer* OR "cancer patient*"	4,718,229
3	Describing the concepts-communication and experiences	
Communication and communication experience	(MH "Communication+") OR (MH "Communication Methods, Total") OR (MH "Disclosure+") OR communication OR "information exchange" OR "communication pattern*" OR "communication strategy*" OR "communication method*" OR	3,060,614

	"communication technique*" OR disclosure OR interaction* OR experience*	
4	Combining all concepts	
	1 AND 2 AND 3	9,478
5	Limiter 1: Date between 2012 and June 2022	4,962
6	Limiter 2: adult 19 years and above	2,393
7	Limiter 3: English publication	2,317

CINAHL

1	Describing the population of this study	Results
Participants	(MH "Nurses+") OR (MH "Nursing Care+") OR “nursing staff” OR nurse* OR "nursing care" OR "nurse practitioner*" OR "hospital nurse*" OR "non-speciali#ed nurse*."	741,262
2	Describing the concepts –Cancers	
Cancers	(MH "Neoplasms+") OR (MH "Cancer Patients") OR neoplasm* OR neoplasia Or tum#r* OR “Malignant neoplasm*” OR	795,776

	Malignan* OR cancer* OR “cancer patients”	
3	Describing the concepts-communication and experiences	
Communication and communication experience	(MH "Communication+") OR (MH "Communication Methods, Total") OR (MH "Disclosure+") OR communication OR “information exchange” OR “communication pattern*” OR “communication strategy*" OR "communication method*" OR “communication technique*” OR disclosure OR interaction* OR experience*	895,801
4	Combining all concepts	
	1 AND 2 AND 3	9,442
5	Limiter 1: Date between 2012 and June 2022	5,188
6	Limiter 2: all adults (18 and above)	1,940
7	Limiter 3: English publication	1,864

PsycINFO

1	Describing the population of this study	Results
Participants	MM "Nursing" OR DE "Nurses" OR DE "Psychiatric Nurses" OR DE "Public Health Service Nurses" OR DE "School Nurses" OR “nursing staff” OR nurs* OR “nursing care” OR “nurse practitioner*” OR “hospital nurse*” OR "non-speciali#ed nurse*"	111,885
2	Describing the concepts –Cancers	
Cancers	DE "Neoplasms" OR DE "Benign Neoplasms" OR DE "Breast Neoplasms" OR DE "Endocrine Neoplasms" OR DE "Leukemias" OR DE "Melanoma" OR DE "Metastasis" OR DE "Nervous System Neoplasms" OR DE "Terminal Cancer" OR neoplasia OR tum#r* OR “Malignant neoplasm*” OR Malignan* OR cancer* OR cancer patient*	90,760

3	Describing the concepts-communication and experiences	
Communication and communication experience	DE "Communication" OR DE "Augmentative Communication" OR DE "Communication Barriers" OR DE "Electronic Communication" OR DE "Interpersonal Communication" OR DE "Messages" OR DE "Nonverbal Communication" OR DE "Persuasive Communication" OR DE "Privileged Communication" OR DE "Scientific Communication" OR DE "Social Communication" OR DE "Verbal Communication" OR communication OR “information exchange” OR “communication pattern*” OR “communication strategy*" OR "communication method*" OR "communication technique*" OR disclosure OR interaction* OR experience*	1,183,559
4	Combining all concepts	
	1 AND 2 AND 3	2,450

5	Limiter 1: Date between 2012 and June 2022	1,413
6	Limiter 2: adult 18 years and above	1,125
7	Limiter 3: English publication	1,116
