# Supplementary material for: Communication to adult patients undergoing cancer care by non-specialist nurses: a scoping review protocol
Source: BMJ Open. 2024 Mar 20;14(3):e081326. doi: 10.1136/bmjopen-2023-081326 (PMC10961544; doi:10.1136/bmjopen-2023-081326)
Supplement: Author's manuscript [file bmjopen-2023-081326.draft_revisions.pdf]

BMJ Open is committed to open peer review. As part of this commitment we make the peer review history of every article we publish publicly available.

When an article is published we post the peer reviewers' comments and the authors' responses online. We also post the versions of the paper that were used during peer review. These are the versions that the peer review comments apply to.

The versions of the paper that follow are the versions that were submitted during the peer review process. They are not the versions of record or the final published versions. They should not be cited or distributed as the published version of this manuscript.

BMJ Open is an open access journal and the full, final, typeset and author-corrected version of record of the manuscript is available on our site with no access controls, subscription charges or pay-per-view fees (http://bmjopen.bmj.com).

If you have any questions on BMJ Open's open peer review process please email info.bmjopen@bmj.com

BMJ Open

Communication to Adult Patients Undergoing Cancer Care by Non-Specialist Nurses: a Scoping Review Protocol

Journal:	*BMJ Open*
Manuscript ID	bmjopen-2023-081326
Article Type:	Protocol
Date Submitted by the Author:	24-Oct-2023
Complete List of Authors:	Kachimanga, Chiyembekezo; University of Stirling; Partners In Health Malawi<br>McGlashan, Jennifer; University of Stirling<br>Cunningham, Nicola; University of Stirling,<br>Hoyle, Louise; University of Stirling
Keywords:	ONCOLOGY, Systematic Review, Nursing Care, Nurses

SCHOLARONE™
Manuscripts

Communication to Adult Patients Undergoing Cancer Care by Non-Specialist Nurses: a Scoping Review Protocol

Target journal: BMJ Open

Authors

Chiyembekezo Kachimanga[1,2], Jennifer McGlashan [1], Nicola Cunningham [1], Louise Hoyle[1]

Affiliation

1. University of Stirling
2. Partners In Health Malawi

*Corresponding author

Word count: 2520

For peer review only

For peer review only - http://bmjopen.bmj.com/site/about/guidelines.xhtml

ABSTRACT

Introduction: Little is known regarding how non-specialist nurses communicate with patients living with cancer when receiving care outside their cancer units/teams. This scoping review aims to identify, examine and report on the currently available evidence about communication by non-specialist nurses when caring for adults living with cancer outside of their cancer care unit/teams.

Methods and analysis: A scoping review following the JBI methodology for scoping reviews will be conducted. We will search for empirical studies that meet the inclusion criteria in six databases (MEDLINE, PubMed, CINAHL, Embase, Scopus, and PsycINFO). Hand-searching in references of included articles will be performed to find additional articles. The population of interest will be non-specialist nurses. Three concepts will be explored, namely 1) all adult patients living with cancer, 2) a focus on three stages of the cancer continuum of care (cancer diagnosis, treatment, and survivorship), and 3) a focus on communication between non-specialist nurses and patients living with cancer. We will include studies describing all healthcare settings outside patients' specialised cancer units or oncology teams. After article selection, two reviewers will independently screen titles and abstracts and perform a full-text article review, risk of bias assessments and data extraction. A third reviewer will resolve all disagreements. A narrative summary will provide an overview of how the results relate to the research aims and questions. The included articles will be limited to English and published between 2012 and 2023

Ethics and dissemination: No ethical approval is required since we will use publicly available empirical research sources**.** This review will provide current research on communication by non-specialist nurses with patients with a cancer diagnosis outside

For peer review only - http://bmjopen.bmj.com/site/about/guidelines.xhtml

of an oncology setting, evidence that will support effective communication. As such, we aim to disseminate the findings in academic conferences and peer-reviewed journals.

Keywords: cancer, neoplasms, communication, scoping reviews, non-specialist nurses

Strengths and Limitations of this study

1. This will be the first review that provides a comprehensive summary of communication by non-specialist nurses with patients with a cancer diagnosis outside of an oncology setting.
2. The scoping review will be systematic as it will be guided by JBI methodology for scoping reviews and will be reported using the checklist for preferred reporting for systematic reviews and meta-analyses extension for scoping reviews (PRISMA -ScR). Using these frameworks will support transparency and scientific rigour.
3. The review will focus on empirical studies published in academic journals. We will exclude articles published in grey literature.
4. The review will be limited to studies published in English between 2012 and 2023.

For peer review only

For peer review only - http://bmjopen.bmj.com/site/about/guidelines.xhtml

INTRODUCTION

Cancer is one of the most commonly diagnosed diseases and the leading cause of death worldwide(1). In 2020 alone, over 20 million people were diagnosed with cancer, and nearly 17% of all deaths were caused by cancer (2). Cancers affect all regions of the world, and the incidence of cancers will increase by up to 50% by 2040(3).

The higher morbidity and mortality of cancer require appropriate care at all health system levels. Cancer care is provided by a multidisciplinary team from the community to tertiary care facilities. Among the essential healthcare workers are the nurses, the most numerous healthcare workers working with people diagnosed with cancer(4). Whether generalists or specialists, nurses play essential roles in managing patients diagnosed with cancer through screening, treatment, survivorship, or end-of-life care(5). Nurse-led cancer interventions, which include direct patient care, education, counselling and care coordination, improve high-quality patient-centred cancer care(6). Patients with cancer diagnoses need care from a well-trained, updated, and skilled nursing workforce(7).

Effective communication is one of the biggest challenges faced by nurses(8). Challenges with communication increased with the COVID-19 pandemic as nurses faced communicating unprecedented situations to patients and their families(8). Additionally, access to healthcare workers was reduced, and healthcare delivery changed to virtual delivery rather than face-to-face, negatively impacting communication (9). Indeed, communication has been a critical research priority for nurses since the pandemic(5, 8, 10). Patients living with cancer require effective communication from their healthcare providers. Effective communication means providing all necessary information to allow a good understanding of their cancer diagnosis and managing emotional responses

For peer review only - http://bmjopen.bmj.com/site/about/guidelines.xhtml

stemming from an awareness of the diagnosis (11). Effective communication also helps patients with a cancer diagnosis make informed care decisions and fosters strong relationships between patients and their providers (12, 13). Effective communication is beneficial and can improve clinical outcomes and patients' care experience (13). Furthermore, effective communication enhances patient satisfaction and quality of life, reduces providers' stress and burnout, improves adherence to cancer care, and eases caretaker burden(12-17).

Communication with patients by healthcare providers is bi-directional and provided throughout the cancer care continuum(18). The bi-directional nature entails that healthcare workers provide information that allows patients to make decisions based on their understanding of the disease, and patients are empowered to make decisions that will ultimately improve their care(11). The cancer continuum depicts the steps involved in cancer care and control and includes 1) prevention and risk reduction, 2) screening, 3) cancer diagnosis, 4) treatment, 4) survivorship, and 5) end-of-life care (19). Throughout this cancer continuum, communication is provided by all healthcare workers involved in patient care. Communication with patients with a cancer diagnosis does not only happen when patients receive care from their oncology teams but also during any encounter with health care providers, including care outside their oncology teams, emergency settings and non-cancer related clinical visits (20, 21). Regardless of the setting of clinical encounters, communication should be continuous and reinforced over time.

Effective communication is widely recognised as one of the dimensions of quality in cancer care (22). However, despite this recognition, effective communication remains one of the most common unmet needs for patients with cancer (13). Effective

For peer review only - http://bmjopen.bmj.com/site/about/guidelines.xhtml

communication goes beyond providing understandable information; it involves communicating in a patient-centred approach- a process where communication is provided sensitively, with respect to patient's autonomy and involving patients in the decision-making process (23, 24).

Communication in cancer care is a learned clinical skill and goes beyond medical interviews due to the complexity and life-threatening issues commonly discussed with patients living with cancer (25, 26). As such, for oncologists and other healthcare workers working in cancer units, rigorous communication training should be provided as part of their routine training(27, 28). However, even if training is in place, evidence suggests that trained healthcare workers need help with effective communication. For example, a study in cancer care settings in Africa found that only 40% of nurses and 20% of physicians had formal communications training (29). Similar studies in Kenya and Belgium have also shown that lack of communication training is a major challenge in cancer care(30-32).

Patients with a cancer diagnosis may require care outside their usual care teams, and nurses play a more prominent role in this context. Nurses, including non-specialised nurses, are usually first-line workers, spend the most time with clients and interface with cancer patients during outpatient and in-patient services (33, 34). Non-specialist nurses also have essential roles in patient management, including direct nursing care, patient navigation, educating other healthcare workers, and care coordination (35, 36).

To ensure good outcomes, non-specialist nurses must be equipped with practical communication skills when caring for an individual diagnosed with cancer. Little is known regarding how non-specialist nurses communicate with patients living with cancer when receiving care outside their cancer units/teams. This scoping review aims

For peer review only - http://bmjopen.bmj.com/site/about/guidelines.xhtml

to understand non-specialist nurses' roles and skills in effectively communicating with adult patients living with cancer.

Aims

The main objective of this scoping review will be to identify the roles and skills of non-specialist nurses in providing effective communication to adult patients with a diagnosis of cancer receiving any health services outside of their cancer care units. An initial scoping search was conducted in four databases (Prospero, PubMed, JBI systematic review register and Cochrane database) to check if any review is in progress or has been published on this scoping review objective. No review similar to this topic is being conducted or has been published (Appendix 1). This scoping review will answer three main questions:

1. What are the roles of non-specialist nurses in providing effective communication to adult patients with a diagnosis of cancer attending any health service outside of their cancer units?
2. How and when do non-specialist nurses communicate with adult patients with cancer attending any health services outside of cancer units?
3. What communication skills do they have to effectively communicate to adult patients with cancer attending any health services outside of cancer units?

METHODS AND ANALYSIS

To identify, examine and report on the currently available evidence about communication by non-specialist nurses when caring for an individual with a diagnosis of cancer, the most appropriate approach to gathering and synthesising evidence will be a scoping review. This review will be guided by the JBI methodology for scoping reviews

For peer review only - http://bmjopen.bmj.com/site/about/guidelines.xhtml

(37). It will use the checklist for preferring reporting for systematic reviews and meta-analyses extension for scoping reviews (PRISMA -ScR)(38).

Inclusion criteria

To define the scope and inclusion criteria of the review, we have used the population, concepts, and context (PCC) framework (37, 39). The population of interest will be non-specialist nurses. Three concepts will be explored, namely 1) All adult patients (18 years and above) living with cancer, 2) a focus on three stages of the cancer continuum of care (cancer diagnosis, treatment, and survivorship), 3) a focus on communication between non-specialist nurses and patients living with cancer. In context, we will include studies describing all healthcare settings outside patients' specialised cancer units or oncology teams. Table 1 details the inclusion and exclusion criteria.

Inclusion criteria	Exclusion criteria
1) Studies reporting on non-specialist nurses. Non-specialist nurses are not specifically trained to provide expert advice and care for cancer patients and do not have formal certification in cancer care.<br>2) Patients living with cancer aged 18 years and above.<br>3) Patients living with cancer in any of the three stages of the cancer continuum of care (diagnosis, treatment and survivorship)	1) Studies reporting on specialist nurses, student nurses or other health care workers.<br>2) Participants aged less than 18 years.<br>3) Studies reporting on cancer screening, end-of-life care or palliative care stage of cancer continuum of care<br>4) Studies not reporting on communication.

For peer review only - http://bmjopen.bmj.com/site/about/guidelines.xhtml

4) Studies reporting on communication between non-specialist nurses and patients diagnosed with cancer. We will explore the following concepts:<br>a. Experiences and roles of non-specialist nurses in communicating with cancer patients<br>b. Context where communication between non-specialist nurses and cancer patients occurs<br>c. How communication (verbal and non-verbal) occurs between non-specialist nurses and patients with cancer<br>d. Training and skills in communicating with cancer patients by non-specialist nurses<br>5) Communication occurs when cancer patients access services outside of the cancer unit /care teams<br>6) Studies published in the English language<br>7) Studies conducted between 2012 and 2023<br>8) Qualitative, quantitative, or mixed-methods study designs	5) Studies reporting on patients that cannot be able to communicate or have limited capacity to communicate, e.g. Dementia and Alzheimer's disease<br>6) Studies conducted in cancer care units/specialised units<br>7) Studies in other languages other than English<br>8) Studies outside the time limit

For peer review only

For peer review only - http://bmjopen.bmj.com/site/about/guidelines.xhtml

Table 1 Inclusion and exclusion criteria

Sources of evidence

The review will consider empirical studies from peer-reviewed articles that used quantitative, qualitative, and mixed-method designs. All designs within these types of studies will be included. All other study types, including review studies, commentaries, policy-related documents, and conference proceedings, will be excluded. We will also hand-search references of included articles to identify additional articles. As the authors are proficient in the English language only, we will include studies published in the English language only. We also plan to review recent articles on communication; hence, we will restrict studies published in the last decade (Between January 2012 and December 2023).

Search strategy and information sources

This review aims to identify published empirical studies. The search strategy will follow a three-step process as recommended by the JBI methodology for scoping reviews(40). In step 1, an initial limited search was undertaken. The following concepts were used as the initial starting point: "non-specialist nurse" AND Cancer AND communication. Each concept term was expanded by looking at synonyms and identifying common terms used in the literature (27, 41, 42). Using the EBSCOhost platform for Medline, CINAHL and PsycINFO databases, appropriate medical subject headings and free text for each concept were developed (43). The 'expand' option to expand some of the subject headings was used as appropriate. CK designed the initial search terms, which LH validated. Table 2 provides an overview of the sample search terms used at this initial point, and Appendix 2 provides the results of this limited search. The titles, abstract and index terms will be analysed from this initial search to inform the final search strategy.

For peer review only - http://bmjopen.bmj.com/site/about/guidelines.xhtml

1	Describing the population of this study
Participants	(MH "Nursing Staff+") OR (MH "Nurses+") OR (MH "Nursing+") OR (MH "Nursing Care+") OR (MH "Nurse Practitioners+") OR (MH "Nursing Staff, Hospital+") OR "nursing staff" OR nurs* OR "nursing care" OR "nurse practitioner*" OR "hospital nurse*" OR "non-speciali#ed nurse*"
2	Describing the concepts –Cancers
Cancers	(MH "Neoplasms+") OR neoplasm* OR neoplasia Or tum#r* OR "Malignant neoplasm*" OR Malignan* OR cancer* OR "cancer patient*"
3	Describing the concepts-communication and experiences
Communication and communication experience	(MH "Communication+") OR (MH "Communication Methods, Total") OR (MH "Disclosure+") OR communication OR "information exchange" OR "communication pattern*" OR "communication strategy*" OR "communication method*" OR "communication technique*" OR disclosure OR interaction* OR experience*
4	Combining all concepts
	1 AND 2 AND 3
5	Limiter 1: Date between 2012 and June 2022
6	Limiter 2: adult 18 years and above

For peer review only - http://bmjopen.bmj.com/site/about/guidelines.xhtml

For peer review only

7	Limiter 3: English publication

Table 2 Sample Search Terms

In step 2, we will perform another search using the developed search strategy from the previous step. A comprehensive search in the MEDLINE, PubMed, CINAHL, Embase, Scopus, and PsycINFO databases will be performed. In step 3, additional studies will be supplemented by hand-searching the references of included sources for additional citations.

Study selection

After a comprehensive data search in the selected databases, we will export all articles to EndNote software version 20, where all duplicates will be removed (44). Afterwards, all articles will be exported to Covidence software for article screening, full-text review and data extraction (45). Two reviewers will independently review the title and abstracts to identify articles eligible for full-text review, conduct a full-text review to identify articles eligible for data extraction and perform data extraction based on a piloted data extraction tool. A third reviewer will resolve all disagreements during the title and abstract screening, full-text screening, and data extraction. The full text of retrieved sources that do not meet the inclusion criteria will be excluded, but a note will be made for the reason for exclusion. The searching, screening and selection will be documented using a PRISMA-ScR flow diagram.

Data extraction and assessment of methodological quality

We will document the data based on a standardised data collection tool. Before data collection, the tool will be piloted on a selection of articles eligible for the title and abstract screening (37). Two reviewers will independently perform the extraction,

For peer review only - http://bmjopen.bmj.com/site/about/guidelines.xhtml

followed by a discussion to resolve differences and create the final data collection tool. We plan to extract the following data based on the eligibility criteria:

1) Details of the article: author, year and design
2) Context: country of the study and the context of where care was provided
3) Cancer care continuum
4) Type of cancer communication
5) Experiences and roles of nurses in cancer communication

Despite pre-specifying the type of data we will collect, we will adjust the data that will be collected during data extraction if necessary. All modifications to the data collection will be described during the final report of the scoping review. The final scoping review report will present results in tabular and narrative format.

Although it is not mandatory to perform risk of bias assessment in scoping reviews, we will include risk of bias assessment in this scoping review (46, 47). Appropriate tools will be identified using JBI critical appraisal tools (https://jbi.global/critical-appraisal-tools). Two reviewers will independently perform a risk of bias assessment, and a third reviewer will resolve all disagreements.

Presentation of results

Tables and figures will provide an overview of extracted data relevant to the research questions. A narrative summary will be written to provide an overview of how the results relate to the research aims and questions.

Patient and public involvement

There is no patient or public involvement in this study.

For peer review only - http://bmjopen.bmj.com/site/about/guidelines.xhtml

DISCUSSION

To our knowledge, this will be the first review that looks at communication by non-specialist nurses with patients with a cancer diagnosis outside of an oncology setting. Patients living with cancer require comprehensive information regarding their diagnosis, which is often conveyed during provider-patient communication. While effective communication brings hope, peace of mind and trust in healthcare workers, ineffective communication leads to sub-optimal cancer care, increased patient and caregiver distress and lack of trust in healthcare workers (48).

Understanding the communication between non-specialised nurses and patients with a cancer diagnosis has implications for practice, policy and research. From a practical point of view, this review will highlight gaps in cancer communication, which may inform areas where clinical guidelines and training need to be included. The results may also influence policymakers by introducing narrative evidence on best practices or gaps in cancer communication by non-specialised nurses. Finally, the study will help identify gaps in the literature on cancer communication. Based on these gaps, further studies, including systematic reviews, may be developed to understand specific aspects of cancer communication.

ETHICS AND DISSEMINATION

Ethical approval will not be needed as we will use publicly available published empirical studies. We plan to disseminate the results in nursing specific conferences and other academic conferences. The final report will be published in peer-reviewed academic journal.

For peer review only - http://bmjopen.bmj.com/site/about/guidelines.xhtml

REFERENCES

1. World Health Organization. Cancer 2022 [updated 3 february 2022. Available from: https://www.who.int/news-room/fact-sheets/detail/cancer.
2. Ferlay J, Ervik M, Lam F, Colombet M, Mery L, Piñeros M, et al. Global Cancer Observatory: Cancer Today Lyon, France: International Agency for Research on Cancer; 2020 [Available from: https://gco.iarc.fr/today.
3. Sung H, Ferlay J, Siegel RL, Laversanne M, Soerjomataram I, Jemal A, et al. Global Cancer Statistics 2020: GLOBOCAN Estimates of Incidence and Mortality Worldwide for 36 Cancers in 185 Countries. CA Cancer J Clin. 2021;71(3):209-49.
4. Challinor JM, Alqudimat MR, Teixeira TOA, Oldenmenger WH. Oncology nursing workforce: challenges, solutions, and future strategies. The Lancet Oncology. 2020;21(12):e564-e74.
5. Dowling M, Efstathiou N, Drury A, Semple C, Fernández-Ortega P, Brochstedt Dieperink K, et al. Cancer nursing research priorities: A rapid review. Eur J Oncol Nurs. 2023;63:102272.
6. Charalambous A, Wells M, Campbell P, Torrens C, Östlund U, Oldenmenger W, et al. A scoping review of trials of interventions led or delivered by cancer nurses. International Journal of Nursing Studies. 2018;86:36-43.
7. Young AM, Charalambous A, Owen RI, Njodzeka B, Oldenmenger WH, Alqudimat MR, et al. Essential oncology nursing care along the cancer continuum. Lancet Oncol. 2020;21(12):e555-e63.
8. Bowman BA, Back AL, Esch AE, Marshall N. Crisis Symptom Management and Patient Communication Protocols Are Important Tools for All Clinicians Responding to COVID-19. J Pain Symptom Manage. 2020;60(2):e98-e100.
9. Legge H, Toohey K, Kavanagh PS, Paterson C. The unmet supportive care needs of people affected by cancer during the COVID-19 pandemic: an integrative review. J Cancer Surviv. 2023;17(4):1036-56.
10. Zanville NZ, Cohen B, Gray TF, Phillips J, Linder L, Starkweather A, et al. The Oncology Nursing Society Rapid Review and Research Priorities for Cancer Care in the Context of COVID-19. Oncol Nurs Forum. 2021;48(2):131-45.
11. National Cancer Institute. Communication in Cancer Care 2022 [Available from: https://www.cancer.gov/about-cancer/coping/adjusting-to-cancer/communication-hp-pdq.
12. Diefenbach M, Turner G, Carpenter KM, Sheldon LK, Mustian KM, Gerend MA, et al. Cancer and patient-physician communication. J Health Commun. 2009;14 Suppl 1(Suppl 1):57-65.
13. Li J, Luo X, Cao Q, Lin Y, Xu Y, Li Q. Communication Needs of Cancer Patients and/or Caregivers: A Critical Literature Review. Journal of Oncology. 2020;2020:7432849.
14. Zachariae R, Pedersen CG, Jensen AB, Ehrnrooth E, Rossen PB, von der Maase H. Association of perceived physician communication style with patient satisfaction, distress, cancer-related self-efficacy, and perceived control over the disease. Br J Cancer. 2003;88(5):658-65.
15. Lorusso D, Bria E, Costantini A, Di Maio M, Rosti G, Mancuso A. Patients' perception of chemotherapy side effects: Expectations, doctor-patient communication and impact on quality of life - An Italian survey. Eur J Cancer Care (Engl). 2017;26(2).
16. Jacobs JM, Pensak NA, Sporn NJ, MacDonald JJ, Lennes IT, Safren SA, et al. Treatment Satisfaction and Adherence to Oral Chemotherapy in Patients With Cancer. J Oncol Pract. 2017;13(5):e474-e85.
17. Graham J, Ramirez A. Improving the working lives of cancer clinicians. Eur J Cancer Care (Engl). 2002;11(3):188-92.
18. Krishnasamy M, Hyatt A, Chung H, Gough K, Fitch M. Refocusing cancer supportive care: a framework for integrated cancer care. Support Care Cancer. 2022;31(1):14.
19. Nekhlyudov L. Introduction and Overview. In: Nekhlyudov L, Goel MS, Lin JJ, Overholser L, Peairs KS, editors. Caring for Patients Across the Cancer Care Continuum: Essentials for Primary Care. Cham: Springer International Publishing; 2019. p. 1-5.

For peer review only

For peer review only - http://bmjopen.bmj.com/site/about/guidelines.xhtml

20. Shelburne N, Simonds NI, Jensen RE, Brown J. Cancer-related emergency and urgent care: expanding the research agenda. Emergency Cancer Care. 2022;1(1):4.
21. Heins MJ, Lorenzi MF, Korevaar JC, McBride ML. Non-oncology physician visits after diagnosis of cancer in children. BMC Family Practice. 2016;17(1):60.
22. Ianovici C, Purcărea VL, Gheorghe IR, Blidaru A. The complexity of physician-patient communication and its impact in non-medical fields. A surgical oncology approach. J Med Life. 2023;16(4):631-4.
23. Marron JM, Cronin AM, Kang TI, Mack JW. Intended and unintended consequences: Ethics, communication, and prognostic disclosure in pediatric oncology. Cancer. 2018;124(6):1232-41.
24. Steven B, Lange L, Schulz H, Bleich C. Views of psycho-oncologists, physicians, and nurses on cancer care-A qualitative study. PLoS One. 2019;14(1):e0210325.
25. Back AL, Arnold RM, Baile WF, Tulsky JA, Fryer-Edwards K. Approaching difficult communication tasks in oncology. CA Cancer J Clin. 2005;55(3):164-77.
26. Fleischer S, Berg A, Zimmermann M, Wüste K, Behrens J. Nurse-patient interaction and communication: A systematic literature review. Journal of Public Health. 2009;17(5):339-53.
27. Moore PM, Rivera S, Bravo-Soto GA, Olivares C, Lawrie TA. Communication skills training for healthcare professionals working with people who have cancer. Cochrane Database Syst Rev. 2018;7(7):Cd003751.
28. Gilligan T, Coyle N, Frankel RM, Berry DL, Bohlke K, Epstein RM, et al. Patient-Clinician Communication: American Society of Clinical Oncology Consensus Guideline. J Clin Oncol. 2017;35(31):3618-32.
29. Lounsbury DW, Nichols S, Asuzu C, Odiyo P, Alis A, Qadir M, et al. Communicating bad news to patients and families in African oncology settings. Psychooncology. 2023;32(1):47-57.
30. Henry M, Nichols S, Hwang JM, Nichols SD, Odiyo P, Watson M, et al. Barriers to communicating a cancer diagnosis to patients in a low- to middle-income context. Journal of Psychosocial Oncology Research and Practice. 2021;3(2):e049.
31. Horlait M, Van Belle S, Leys M. The need for adequate communication training programs for palliative care in multidisciplinary teams in oncology settings. International Journal of Integrated Care. 2016.
32. Horlait M, Chambaere K, Pardon K, Deliens L, Van Belle S. What are the barriers faced by medical oncologists in initiating discussion of palliative care? A qualitative study in Flanders, Belgium. Supportive Care in Cancer. 2016;24(9):3873-81.
33. He M, Zhu X, Dong Y, Zhang S, Fang S, Wang W, et al. Exploring the role of communication in missed nursing care: A systematic review. J Adv Nurs. 2022;78(12):4019-33.
34. Kwame A, Petrucka PM. A literature-based study of patient-centered care and communication in nurse-patient interactions: barriers, facilitators, and the way forward. BMC Nurs. 2021;20(1):158.
35. Cooper JM, Loeb SJ, Smith CA. The primary care nurse practitioner and cancer survivorship care. Journal of the American Academy of Nurse Practitioners. 2010;22(8):394-402.
36. Garcia-Vivar C, Elizondo N, Ambrosio L. Primary Care Nursing Is Essential to Fully Implement Survivorship Care Plans for Long-term Cancer Survivors and Their Families. Cancer Nursing. 2019;42(3):177-8.
37. Peters M, Godfrey C, McInerney P, Munn Z, Tricco A, Khalil H. Chapter 11: Scoping Reviews (2020 version). In: Aromataris E, Munn Z, editors. JBI Manual for Evidence Synthesis: JBI; 2020.
38. Tricco AC, Lillie E, Zarin W, O'Brien KK, Colquhoun H, Levac D, et al. PRISMA Extension for Scoping Reviews (PRISMA-ScR): Checklist and Explanation. Ann Intern Med. 2018;169(7):467-73.
39. Lockwood C, dos Santos KB, Pap R. Practical Guidance for Knowledge Synthesis: Scoping Review Methods. Asian Nursing Research. 2019;13(5):287-94.
40. Aromataris E, Riitano D. Systematic Reviews: Constructing a Search Strategy and Searching for Evidence. AJN The American Journal of Nursing. 2014;114(5).

For peer review only - http://bmjopen.bmj.com/site/about/guidelines.xhtml

41. Thorn H, Uhrenfeldt L. Experiences of non-specialist nurses caring for patients and their significant others undergoing transitions during palliative end-of-life cancer care: a systematic review. JBI Database System Rev Implement Rep. 2017;15(6):1711-46.
42. Graetz DE, Garza M, Rodriguez-Galindo C, Mack JW. Pediatric cancer communication in low- and middle-income countries: A scoping review. Cancer. 2020;126(23):5030-9.
43. EBSCO Information Services. EBSCOhost Research Platform 2023 [Available from: https://www.ebsco.com/products/ebscohost-research-platform.
44. Clarivate. EndNote 20 2023 [
45. Veritas Health Innovation. Covidence systematic review software, Melbourne, Australia.2023 [Available from: Available at www.covidence.org.
46. Peterson J, Pearce PF, Ferguson LA, Langford CA. Understanding scoping reviews: Definition, purpose, and process. J Am Assoc Nurse Pract. 2017;29(1):12-6.
47. Pollock D, Davies EL, Peters MDJ, Tricco AC, Alexander L, McInerney P, et al. Undertaking a scoping review: A practical guide for nursing and midwifery students, clinicians, researchers, and academics. Journal of Advanced Nursing. 2021;77(4):2102-13.
48. Collet R, Major M, van Egmond M, van der Leeden M, Maccow R, Eskes A, et al. Experiences of interaction between people with cancer and their healthcare professionals: A systematic review and meta-synthesis of qualitative studies. Eur J Oncol Nurs. 2022;60:102198.

AUTHORS' CONTRIBUTION

CK conceptualised the study, developed the methods, developed the preliminary search strategy, wrote the first draft, critically reviewed the draft manuscript and approved the final draft for submission.

LH conceptualised the study, developed the methods, developed the preliminary search strategy, critically reviewed the draft manuscript and approved the final draft for submission.

JM conceptualised the study, critically reviewed the draft manuscript and approved the final draft for submission.

NC provided critical inputs to methods and editing of this protocol, critically reviewed the draft manuscript and approved the final draft for submission.

COMPETING INTERESTS

For peer review only

For peer review only - http://bmjopen.bmj.com/site/about/guidelines.xhtml

Non declared.

FUNDING

This research received no specific grant from any funding agency in the public, commercial or not-for-profit sector.

For peer review only

For peer review only - http://bmjopen.bmj.com/site/about/guidelines.xhtml

For peer review only

For peer review only - http://bmjopen.bmj.com/site/about/guidelines.xhtml

Appendix 1 Scoping search results

On 24 October 2022, a scoping search was conducted to check if this scoping review (or any review) is in process or has been conducted in the past. JBI systematic review register, Prospero, Cochrane register, and PubMed was searched using the simple search term "cancer communication". The table below summarises the findings:

Database	Summary
Prospero	The search retrieved nine records. No registered review of the proposed topic.
JBI systematic review register	Only three articles were retrieved. No article included cancer communication.
PubMed	After filtering by meta-analysis, reviews, and systematic reviews, the search term "cancer communication*" yielded 136 studies. No review was similar to this scoping review objectives.
Cochrane	The search retrieved 35 records. No review was similar to this scoping review objectives.

For peer review only

Appendix 2 Initial data extraction

MEDLINE

1	Describing the population of this study	Results
Participants	(MH "Nursing Staff+") OR (MH "Nurses+") OR (MH "Nursing+") OR (MH "Nursing Care+") OR (MH "Nurse Practitioners+") OR (MH "Nursing Staff, Hospital+") OR "nursing staff" OR nurs* OR "nursing care" OR "nurse practitioner*" OR "hospital nurse*" OR "non-speciali#ed nurse*"	633,365
2	Describing the concepts –Cancers	
Cancers	(MH "Neoplasms+") OR neoplasm* OR neoplasia Or tum#r* OR "Malignant neoplasm*" OR Malignan* OR cancer* OR "cancer patient*"	4,718,229
3	Describing the concepts-communication and experiences	
Communication and communication experience	(MH "Communication+") OR (MH "Communication Methods, Total") OR (MH "Disclosure+") OR communication OR "information exchange" OR "communication pattern*" OR "communication strategy*" OR "communication method*" OR	3,060,614

For peer review only

	"communication technique*" OR disclosure OR interaction* OR experience*	
4	Combining all concepts	
	1 AND 2 AND 3	9,478
5	Limiter 1: Date between 2012 and June 2022	4,962
6	Limiter 2: adult 19 years and above	2,393
7	Limiter 3: English publication	2,317

CINAHL

1	Describing the population of this study	Results
Participants	(MH "Nurses+") OR (MH "Nursing Care+") OR “nursing staff” OR nurse* OR "nursing care" OR "nurse practitioner*" OR "hospital nurse*" OR "non-speciali#ed nurse*."	741,262
2	Describing the concepts –Cancers	
Cancers	(MH "Neoplasms+") OR (MH "Cancer Patients") OR neoplasm* OR neoplasia Or tum#r* OR “Malignant neoplasm*” OR	795,776

For peer review only

	Malignan* OR cancer* OR "cancer patients"	
3	Describing the concepts-communication and experiences	
Communication and communication experience	(MH "Communication+") OR (MH "Communication Methods, Total") OR (MH "Disclosure+") OR communication OR "information exchange" OR "communication pattern*" OR "communication strategy*" OR "communication method*" OR "communication technique*" OR disclosure OR interaction* OR experience*	895,801
4	Combining all concepts	
	1 AND 2 AND 3	9,442
5	Limiter 1: Date between 2012 and June 2022	5,188
6	Limiter 2: all adults (18 and above)	1,940
7	Limiter 3: English publication	1,864

For peer review only

PsycINFO

1	Describing the population of this study	Results
Participants	MM "Nursing" OR DE "Nurses" OR DE "Psychiatric Nurses" OR DE "Public Health Service Nurses" OR DE "School Nurses" OR "nursing staff" OR nurs* OR "nursing care" OR "nurse practitioner*" OR "hospital nurse*" OR "non-speciali#ed nurse*"	111,885
2	Describing the concepts –Cancers	
Cancers	DE "Neoplasms" OR DE "Benign Neoplasms" OR DE "Breast Neoplasms" OR DE "Endocrine Neoplasms" OR DE "Leukemias" OR DE "Melanoma" OR DE "Metastasis" OR DE "Nervous System Neoplasms" OR DE "Terminal Cancer" OR neoplasia OR tum#r* OR "Malignant neoplasm*" OR Malignan* OR cancer* OR cancer patient*	90,760

For peer review only

3	Describing the concepts-communication and experiences	
Communication and communication experience	DE "Communication" OR DE "Augmentative Communication" OR DE "Communication Barriers" OR DE "Electronic Communication" OR DE "Interpersonal Communication" OR DE "Messages" OR DE "Nonverbal Communication" OR DE "Persuasive Communication" OR DE "Privileged Communication" OR DE "Scientific Communication" OR DE "Social Communication" OR DE "Verbal Communication" OR communication OR “information exchange” OR “communication pattern*” OR “communication strategy*" OR "communication method*" OR "communication technique*" OR disclosure OR interaction* OR experience*	1,183,559
4	Combining all concepts	
	1 AND 2 AND 3	2,450

For peer review only

5	Limiter 1: Date between 2012 and June 2022	1,413
6	Limiter 2: adult 18 years and above	1,125
7	Limiter 3: English publication	1,116

For peer review only

PRISMA-P 2015 Checklist

This checklist has been adapted for use with protocol submissions to *Systematic Reviews* from Table 3 in Moher D et al: Preferred reporting items for systematic review and meta-analysis protocols (PRISMA-P) 2015 statement. *Systematic Reviews* 2015 **4**:1

Section/topic	#	Checklist item	Information reported		Line number(s)
			Yes	No	
**ADMINISTRATIVE INFORMATION**					
**Title**					
Identification	1a	Identify the report as a protocol of a systematic review	☒	☐	Check title
Update	1b	If the protocol is for an update of a previous systematic review, identify as such	☐	☐	NA
**Registration**	2	If registered, provide the name of the registry (e.g., PROSPERO) and registration number in the Abstract	☐	☒	
**Authors**					
Contact	3a	Provide name, institutional affiliation, and e-mail address of all protocol authors; provide physical mailing address of corresponding author	☒	☐	
Contributions	3b	Describe contributions of protocol authors and identify the guarantor of the review	☒	☐	Check authors contribution
**Amendments**	4	If the protocol represents an amendment of a previously completed or published protocol, identify as such and list changes; otherwise, state plan for documenting important protocol amendments	☐	☐	NA
**Support**					
Sources	5a	Indicate sources of financial or other support for the review	☒	☐	Check funding statement
Sponsor	5b	Provide name for the review funder and/or sponsor	☐	☐	NA
Role of sponsor/funder	5c	Describe roles of funder(s), sponsor(s), and/or institution(s), if any, in developing the protocol	☐	☐	NA
**INTRODUCTION**					
**Rationale**	6	Describe the rationale for the review in the context of what is already known	☒	☐	Introduction section
**Objectives**	7	Provide an explicit statement of the question(s) the review will address with reference to participants, interventions, comparators, and outcomes (PICO)	☒	☐	Aims subsection

BioMed Central
The Open Access Publisher

Section/topic	#	Checklist item	Information reported Yes	Information reported No	Line number(s)
**METHODS**					
**Eligibility criteria**	8	Specify the study characteristics (e.g., PICO, study design, setting, time frame) and report characteristics (e.g., years considered, language, publication status) to be used as criteria for eligibility for the review	☒	☐	Check inclusion criteria and table 1
**Information sources**	9	Describe all intended information sources (e.g., electronic databases, contact with study authors, trial registers, or other grey literature sources) with planned dates of coverage	☒	☐	Check sources of evidence and search strategy and information sources section
**Search strategy**	10	Present draft of search strategy to be used for at least one electronic database, including planned limits, such that it could be repeated	☒	☐	Check search strategy and information sources and table 2
STUDY RECORDS					
Data management	11a	Describe the mechanism(s) that will be used to manage records and data throughout the review	☒	☐	Check data extraction and assessment of the methodological quality section.
Selection process	11b	State the process that will be used for selecting studies (e.g., two independent reviewers) through each phase of the review (i.e., screening, eligibility, and inclusion in meta-analysis)	☒	☐	Check study selection
Data collection process	11c	Describe planned method of extracting data from reports (e.g., piloting forms, done independently, in duplicate), any processes for obtaining and confirming data from investigators	☒	☐	Check presentation of results section
**Data items**	12	List and define all variables for which data will be sought (e.g., PICO items, funding sources), any pre-planned data assumptions and simplifications	☒	☐	Check Data extraction and assessment of methodological quality section
**Outcomes and prioritization**	13	List and define all outcomes for which data will be sought, including prioritization of main and additional outcomes, with rationale	☒	☐	Check Data extraction and assessment of

For peer review only

BioMed Central The Open Access Publisher

Section/topic	#	Checklist item	Information reported Yes	No	Line number(s)
					methodological quality section
**Risk of bias in individual studies**	14	Describe anticipated methods for assessing risk of bias of individual studies, including whether this will be done at the outcome or study level, or both; state how this information will be used in data synthesis	☒	☐	Check Data extraction and assessment of methodological quality section
DATA					
**Synthesis**	15a	Describe criteria under which study data will be quantitatively synthesized	☐	☐	NA
	15b	If data are appropriate for quantitative synthesis, describe planned summary measures, methods of handling data, and methods of combining data from studies, including any planned exploration of consistency (e.g., I^2, Kendall's tau)	☐	☐	NA
	15c	Describe any proposed additional analyses (e.g., sensitivity or subgroup analyses, meta-regression)	☐	☐	NA
	15d	If quantitative synthesis is not appropriate, describe the type of summary planned	☒	☐	Check Data extraction and assessment of methodological quality section
**Meta-bias(es)**	16	Specify any planned assessment of meta-bias(es) (e.g., publication bias across studies, selective reporting within studies)	☐	☐	NA
**Confidence in cumulative evidence**	17	Describe how the strength of the body of evidence will be assessed (e.g., GRADE)	☐	☐	NA

BioMed Central The Open Access Publisher

BMJ Open

Communication to Adult Patients Undergoing Cancer Care by Non-Specialist Nurses: a Scoping Review Protocol

Journal:	*BMJ Open*
Manuscript ID	bmjopen-2023-081326.R1
Article Type:	Protocol
Date Submitted by the Author:	14-Feb-2024
Complete List of Authors:	Kachimanga, Chiyembekezo; University of Stirling<br>McGlashan, Jennifer; University of Stirling<br>Cunningham, Nicola; University of Stirling,<br>Hoyle, Louise; University of Stirling
**Primary Subject Heading**:	Nursing
Secondary Subject Heading:	Oncology, Communication
Keywords:	ONCOLOGY, Systematic Review, Nursing Care, Nurses

SCHOLARONE™
Manuscripts

Communication to Adult Patients Undergoing Cancer Care by Non-Specialist Nurses: a Scoping Review Protocol

Target journal: BMJ Open

Authors

Chiyembekezo Kachimanga[1], Jennifer McGlashan [1], Nicola Cunningham [1], Louise Hoyle[1]

Affiliation

1. University of Stirling

*Corresponding author

Word count: 2753

For peer review only - http://bmjopen.bmj.com/site/about/guidelines.xhtml

ABSTRACT

Introduction: Little is known regarding how non-specialist nurses communicate with patients living with cancer when the patients are receiving care outside of their cancer units/teams. This scoping review aims to identify, examine and report on the currently available evidence about communication by non-specialist nurses when caring for adults living with cancer outside of their cancer care unit/teams.

Methods and analysis: A scoping review following the JBI methodology for scoping reviews will be conducted. We will search for empirical studies that meet the inclusion criteria in six databases (MEDLINE, PubMed, CINAHL, Embase, Scopus, and PsycINFO). Hand-searching in references of included articles will be performed to find additional articles. The population of interest will be non-specialist nurses. Three concepts will be explored, namely 1) all adult patients living with cancer, 2) a focus on three stages of the cancer continuum of care (cancer diagnosis, treatment, and survivorship), and 3) a focus on communication between non-specialist nurses and patients living with cancer. We will include studies describing all healthcare settings outside patients' specialised cancer units or oncology teams. After article selection, two reviewers will independently screen titles and abstracts and perform a full-text article review, risk of bias assessments and data extraction. A third reviewer will resolve all disagreements. A narrative summary will provide an overview of how the results relate to the research aims and questions. The included articles will be limited to English and published between 2012 and 2023

Ethics and dissemination: No ethical approval is required since we will use publicly available empirical research sources**.** This review will provide current research on communication by non-specialist nurses with patients with a cancer diagnosis outside

For peer review only - http://bmjopen.bmj.com/site/about/guidelines.xhtml

of an oncology setting, evidence that will support effective communication. As such, we aim to disseminate the findings in academic conferences and peer-reviewed journals.

Keywords: cancer, neoplasms, communication, scoping reviews, non-specialist nurses

Strengths and Limitations of this study

1. This will be the first review that provides a comprehensive summary of communication by non-specialist nurses with patients with a cancer diagnosis outside of an oncology setting.
2. The scoping review will be systematic and transparent as it will be guided by JBI methodology for scoping reviews and will be reported using the checklist for preferred reporting for systematic reviews and meta-analyses extension for scoping reviews (PRISMA -ScR).
3. The review will only focus on empirical studies published in academic journals and will exclude articles published in grey literature.
4. The review will be limited to studies published in English between 2012 and 2023.

For peer review only

INTRODUCTION

Cancer is one of the most commonly diagnosed non-communicable diseases and the leading cause of death worldwide(1). In 2020 alone, over 20 million people were diagnosed with cancer, and nearly 17% of all deaths were caused by cancer (2). Cancers affect all regions of the world, and the incidence of cancers will increase by up to 50% by 2040(3).

The higher morbidity and mortality of cancer require appropriate care at all health system levels. Cancer care is provided by a multidisciplinary team from the community to tertiary care facilities. Among the essential healthcare providers are the nurses, the most numerous healthcare providers working with people diagnosed with cancer(4). Whether generalists or specialists, nurses play essential roles in managing patients diagnosed with cancer through screening, treatment, survivorship, or end-of-life care(5). Nurse-led cancer interventions, which include direct patient care, education, counselling and care coordination, improve high-quality patient-centred cancer care(6). Patients with cancer diagnoses need care from a well-trained, updated, and skilled nursing workforce(7).

Patients living with cancer require effective communication from their healthcare providers. Effective communication means providing all necessary information to allow a good understanding of their cancer diagnosis and managing emotional responses stemming from an awareness of the diagnosis (8). Effective communication also helps patients with a cancer diagnosis make informed care decisions and fosters strong relationships between patients and their providers (9). Effective communication is beneficial and can improve clinical outcomes and patients' care experience (10). Furthermore, effective communication enhances patient satisfaction and quality of life,

For peer review only - http://bmjopen.bmj.com/site/about/guidelines.xhtml

reduces providers' stress and burnout, improves adherence to cancer care, and eases caretaker burden(9, 11, 12).

Communication with patients by healthcare providers is bi-directional and provided throughout the cancer care continuum(13). The bi-directional nature entails that healthcare providers provide information that allows patients to make decisions based on their understanding of the disease, and patients are empowered to make decisions that will ultimately improve their care(8). The cancer continuum depicts the steps involved in cancer care and control and includes 1) prevention and risk reduction, 2) screening, 3) cancer diagnosis, 4) treatment, 4) survivorship, and 5) end-of-life care (14). Throughout this cancer continuum, communication is provided by all healthcare providers involved in patient care. Communication with patients with a cancer diagnosis does not only happen when patients receive care from their oncology teams but also during any encounter with health care providers, including care outside their oncology teams, emergency settings and non-cancer related clinical visits (15, 16). Regardless of the setting of clinical encounters, communication should be continuous and reinforced over time.

Effective communication is widely recognised as one of the dimensions of quality in cancer care (17). However, despite this recognition, effective communication remains one of the most common unmet needs for patients with cancer (9). Effective communication goes beyond providing understandable information; it involves communicating in a patient-centred approach- a process where communication is provided sensitively, with respect to patient's autonomy and involving patients in the decision-making process (18, 19).

For peer review only

Communication in cancer care is a learned clinical skill and goes beyond medical interviews due to the complexity and life-threatening issues commonly discussed with patients living with cancer (20). As such, for oncologists and other healthcare providers working in cancer units, rigorous communication training should be provided as part of their initial and routine training(21, 22). However, even if communication training is in place, evidence suggests that trained healthcare providers need help with effective communication. For example, a study in cancer care settings in Africa found that only 40% of nurses and 20% of physicians had formal communications training (23). Similar studies in Kenya and Belgium have also shown that lack of communication training is a major challenge in cancer care(24-26). In these studies, emphasis was placed on expanding and improving communication training during pre-service training, providing further guidance and mentorship during cancer care coordination and adapting communication to different contexts and/or cultural backgrounds.

Patients with a cancer diagnosis may require care outside their usual care teams, and nurses play a more prominent role in this context. Nurses, including non-specialised nurses, are usually the first-line workers, they spend the most of their time with clients and interface with cancer patients during outpatient and in-patient services (27, 28). Non-specialist nurses also have essential roles in cancer care which includes: direct nursing care, patient navigation, educating other healthcare providers, and care coordination (29).

To ensure good outcomes, non-specialist nurses must be equipped with practical communication skills when caring for an individual diagnosed with cancer. Little is known regarding how non-specialist nurses communicate with patients living with cancer when receiving care outside their cancer units/teams. This scoping review aims

For peer review only - http://bmjopen.bmj.com/site/about/guidelines.xhtml

to understand non-specialist nurses' roles and skills in effectively communicating with adult patients living with cancer.

Aims

The main objective of this scoping review will be to identify the roles and skills of non-specialist nurses in providing effective communication to adult patients with a diagnosis of cancer receiving any health services outside of their cancer care units. An initial scoping search was conducted in four databases (Prospero, PubMed, JBI systematic review register and Cochrane database) to check if any review is in progress or has been published on this scoping review objective. No review similar to this topic is being conducted or has been published (Appendix 1). This scoping review will answer four main questions:

1. What are the roles of non-specialist nurses in providing effective communication to adult patients with a diagnosis of cancer attending any health service outside of their cancer units?
2. When do non-specialist nurses communicate with adult patients with cancer attending any health services outside of cancer units?
3. How do non-specialist nurses communicate with adult patients with cancer attending any health services outside of cancer units?
4. What communication skills do they have to effectively communicate to adult patients with cancer attending any health services outside of cancer units?

METHODS AND ANALYSIS

To identify, examine and report on the currently available evidence about communication by non-specialist nurses when caring for an individual with a diagnosis of cancer, the most appropriate approach to gathering and synthesising evidence will be

For peer review only - http://bmjopen.bmj.com/site/about/guidelines.xhtml

a scoping review. This review will be guided by the JBI methodology for scoping reviews (30). The JBI methodology for scoping reviews was advanced and refined from two previous scoping review guidance from Arksey and O'Malley(31) and Levac et al.(32). Furthermore, will use the checklist for preferred reporting for systematic reviews and meta-analyses extension for scoping reviews (PRISMA -ScR)(33). We registered the protocol in Open Science Framework registry (https://osf.io/3pmt9)

Inclusion criteria

To define the scope and inclusion criteria of the review, we have used the population, concepts, and context (PCC) framework (30, 34). The population of interest will be non-specialist nurses. Three concepts will be explored, namely 1) All adult patients (18 years and above) living with cancer, 2) a focus on three stages of the cancer continuum of care (cancer diagnosis, treatment, and survivorship), 3) a focus on communication between non-specialist nurses and patients living with cancer. In context, we will include studies conducted in any geographical location describing all healthcare settings outside patients' specialised cancer units or oncology teams. Table 1 details the inclusion and exclusion criteria.

For peer review only

Inclusion criteria	Exclusion criteria
1) Studies reporting on non-specialist nurses. Non-specialist nurses are not specifically trained to provide expert advice and care for cancer patients and do not have formal certification in cancer care.<br>2) Patients living with cancer aged 18 years and above.<br>3) Patients living with cancer in any of the three stages of the cancer continuum of care (diagnosis, treatment and survivorship)<br>4) Studies reporting on communication between non-specialist nurses and patients diagnosed with cancer. We will explore the following concepts:<br>a. Experiences and roles of non-specialist nurses in communicating with cancer patients<br>b. Context where communication between non-specialist nurses and cancer patients occurs	1) Studies reporting on specialist nurses, student nurses or other health care workers.<br>2) Studies reporting on cancer screening, end-of-life care or palliative care stage of cancer continuum of care<br>3) Studies not reporting on communication.<br>4) Studies reporting on patients that cannot be able to communicate or have limited capacity to communicate, e.g. Dementia and Alzheimer's disease.

For peer review only

c. How communication (verbal and non-verbal) occurs between non-specialist nurses and patients with cancer<br>d. Training and skills in communicating with cancer patients by non-specialist nurses<br>5) Communication occurs when cancer patients access services outside of the cancer unit /care teams<br>6) Studies published in the English language<br>7) Studies conducted between 2012 and 2023<br>8) Qualitative, quantitative, or mixed-methods study designs	

Table 1 Inclusion and exclusion criteria

Sources of evidence

The review will consider empirical studies from peer-reviewed articles that used quantitative, qualitative, and mixed-method designs. All designs within these types of studies will be included. All other study types, including review studies, commentaries, policy-related documents, and conference proceedings, will be excluded. We will also hand-search references of included articles to identify additional articles. As the authors are proficient in the English language only, we will include studies published in the English language only. We also plan to review recent articles on communication; hence,

For peer review only

For peer review only - http://bmjopen.bmj.com/site/about/guidelines.xhtml

we will restrict studies published in the last decade (Between January 2012 and December 2023).

Search strategy and information sources

This review aims to identify published empirical studies. The search strategy will follow a three-step process as recommended by the JBI methodology for scoping reviews(35). In step 1, an initial limited search was undertaken in October 2022. The following concepts were used as the initial starting point: "non-specialist nurse" AND Cancer AND communication. Each concept term was expanded by looking at synonyms and identifying common terms used in the literature (21, 36, 37). Using the EBSCOhost platform for Medline, CINAHL and PsycINFO databases, appropriate medical subject headings and free text for each concept were developed (38). The 'expand' option to expand some of the subject headings was used as appropriate. CK designed the initial search terms, which LH validated. Table 2 provides an overview of the sample search terms used at this initial point, and Appendix 2 provides the results of this limited search. The titles, abstract and index terms will be analysed from this initial search to inform the final search strategy.

For peer review only - http://bmjopen.bmj.com/site/about/guidelines.xhtml

1	Describing the population of this study
Participants	(MH "Nursing Staff+") OR (MH "Nurses+") OR (MH "Nursing+") OR (MH "Nursing Care+") OR (MH "Nurse Practitioners+") OR (MH "Nursing Staff, Hospital+") OR "nursing staff" OR nurs* OR "nursing care" OR "nurse practitioner*" OR "hospital nurse*" OR "non-speciali#ed nurse*"
2	Describing the concepts –Cancers
Cancers	(MH "Neoplasms+") OR neoplasm* OR neoplasia Or tum#r* OR "Malignant neoplasm*" OR Malignan* OR cancer* OR "cancer patient*"
3	Describing the concepts-communication and experiences
Communication and communication experience	(MH "Communication+") OR (MH "Communication Methods, Total") OR (MH "Disclosure+") OR communication OR "information exchange" OR "communication pattern*" OR "communication strategy*" OR "communication method*" OR "communication technique*" OR disclosure OR interaction* OR experience*
4	Combining all concepts
	1 AND 2 AND 3
5	Limiter 1: Date between 2012 and June 2022

For peer review only

6	Limiter 2: adult 18 years and above
7	Limiter 3: English publication

Table 2 Sample Search Terms

In step 2, we will perform another search using the developed search strategy from the previous step. A comprehensive search in the MEDLINE, PubMed, CINAHL, Embase, Scopus, and PsycINFO databases will be performed. In step 3, additional studies will be supplemented by hand-searching the references of included sources for additional citations.

Study selection

After a comprehensive data search in the selected databases, we will export all articles to EndNote software version 20, where all duplicates will be removed (39). Afterwards, all articles will be exported to Covidence software for article screening, full-text review and data extraction (40). Two reviewers will independently review the title and abstracts to identify articles eligible for full-text review, conduct a full-text review to identify articles eligible for data extraction and perform data extraction based on a piloted data extraction tool. A third reviewer will resolve all disagreements during the title and abstract screening, full-text screening, and data extraction. The full text of retrieved sources that do not meet the inclusion criteria will be excluded, but a note will be made for the reason for exclusion. The searching, screening and selection will be documented using a PRISMA-ScR flow diagram (figure 1). The flow diagram will be updated once the review has been completed.

Figure 1 PRISMA flow diagram

NB * The search results will be update by the end of the review

For peer review only - http://bmjopen.bmj.com/site/about/guidelines.xhtml

Data extraction and assessment of methodological quality

We will document the data based on a standardised data collection tool. Before data collection, the tool will be piloted on a selection of articles eligible for the title and abstract screening (30). Two reviewers will independently perform the extraction, followed by a discussion to resolve differences and create the final data collection tool (see table 3 for sample data collection tool). We plan to extract the following data based on the eligibility criteria:

1) Details of the article: author, year, and study design
2) Context: country of the study and health care setting
3) Cancer care continuum
4) Types and roles played by non-specialist nurses in cancer care
5) Timing, frequency and contexts of cancer communication
6) Communication methods used
7) Communication training and on-going skills (pre-service and in-service skills) acquired by non-specialist nurses.

Despite pre-specifying the type of data we will collect, we will adjust the data that will be collected during data extraction if necessary. All modifications to the data collection will be described during the final report of the scoping review.

For peer review only - http://bmjopen.bmj.com/site/about/guidelines.xhtml

Author, Year	Study design	Participants	Context	Cancer continuum of care	Type/roles of non-specialist nurses	Timing of cancer communication	Communication methods	Communication training/skills

Table 3 Sample data collection tool

Although it is not mandatory to perform risk of bias assessment in scoping reviews, we will include risk of bias assessment in this scoping review (41, 42). Risk of bias assessment is an appraisal of the methodological quality of the included studies in-order to inform the synthesis of the collected data(43). In this review, we will use risk of bias assessment to identify studies that should be included with the purpose of including studies with low risk of biases. Appropriate tools will be identified using JBI critical appraisal tools (https://jbi.global/critical-appraisal-tools). Two reviewers will independently perform a risk of bias assessment, and a third reviewer will resolve all disagreements.

Presentation of results and expert consultation.

We plan to map the evidence based on PCC using both quantitative and qualitative study designs. For quantitative designs, a narrative summary will be written to provide an overview of how the results relate to the research aims and questions. For qualitative designs, descriptive content analysis will be used. Tables and figures will be used to provide an overview of extracted data relevant to the research questions. We plan to

For peer review only - http://bmjopen.bmj.com/site/about/guidelines.xhtml

continue the comprehensive literature search in April 2024 and finalise the review by March 2025.

During the comprehensive literature search and data extraction we will seek additional support from subject-specific experts. We will seek an expert information specialist to validate the final search strategy. We will also seek support from a cancer expert to validate the findings prior to final dissemination.

Patient and public involvement

There is no patient or public involvement in this study.

DISCUSSION

To our knowledge, this will be the first review that looks at communication by non-specialist nurses with patients with a cancer diagnosis outside of an oncology setting. Patients living with cancer require comprehensive information regarding their diagnosis, which is often conveyed during provider-patient communication. While effective communication brings hope, peace of mind and trust in healthcare providers, ineffective communication leads to sub-optimal cancer care, increased patient and caregiver distress and lack of trust in healthcare providers (20).

Understanding the communication between non-specialised nurses and patients with a cancer diagnosis has implications for practice, policy and research. From a practical point of view, this review will highlight gaps in cancer communication, which may inform areas where clinical guidelines and training need to be included. The results may also influence policymakers by introducing narrative evidence on best practices or gaps in cancer communication by non-specialised nurses. Finally, the study will help identify gaps in the literature on cancer communication. Based on these gaps, further studies,

For peer review only - http://bmjopen.bmj.com/site/about/guidelines.xhtml

including systematic reviews, may be developed to understand specific aspects of cancer communication.

ETHICS AND DISSEMINATION

Ethical approval will not be needed as we will use publicly available published empirical studies. We plan to disseminate the results in nursing specific conferences and other academic conferences. The final report will be published in peer-reviewed academic journal.

For peer review only

For peer review only - http://bmjopen.bmj.com/site/about/guidelines.xhtml

REFERENCES

1. World Health Organization. Cancer 2022 [updated 3 february 2022. Available from: https://www.who.int/news-room/fact-sheets/detail/cancer.
2. Ferlay J, Ervik M, Lam F, Colombet M, Mery L, Piñeros M, et al. Global Cancer Observatory: Cancer Today Lyon, France: International Agency for Research on Cancer; 2020 [Available from: https://gco.iarc.fr/today.
3. Sung H, Ferlay J, Siegel RL, Laversanne M, Soerjomataram I, Jemal A, et al. Global Cancer Statistics 2020: GLOBOCAN Estimates of Incidence and Mortality Worldwide for 36 Cancers in 185 Countries. CA Cancer J Clin. 2021;71(3):209-49.
4. Challinor JM, Alqudimat MR, Teixeira TOA, Oldenmenger WH. Oncology nursing workforce: challenges, solutions, and future strategies. The Lancet Oncology. 2020;21(12):e564-e74.
5. Dowling M, Efstathiou N, Drury A, Semple C, Fernández-Ortega P, Brochstedt Dieperink K, et al. Cancer nursing research priorities: A rapid review. Eur J Oncol Nurs. 2023;63:102272.
6. Charalambous A, Wells M, Campbell P, Torrens C, Östlund U, Oldenmenger W, et al. A scoping review of trials of interventions led or delivered by cancer nurses. International Journal of Nursing Studies. 2018;86:36-43.
7. Young AM, Charalambous A, Owen RI, Njodzeka B, Oldenmenger WH, Alqudimat MR, et al. Essential oncology nursing care along the cancer continuum. Lancet Oncol. 2020;21(12):e555-e63.
8. National Cancer Institute. Communication in Cancer Care 2022 [Available from: https://www.cancer.gov/about-cancer/coping/adjusting-to-cancer/communication-hp-pdq.
9. Li J, Luo X, Cao Q, Lin Y, Xu Y, Li Q. Communication Needs of Cancer Patients and/or Caregivers: A Critical Literature Review. Journal of Oncology. 2020;2020:7432849.
10. Lafata JE, Shay LA, Winship JM. Understanding the influences and impact of patient-clinician communication in cancer care. Health Expect. 2017;20(6):1385-92.
11. Lorusso D, Bria E, Costantini A, Di Maio M, Rosti G, Mancuso A. Patients' perception of chemotherapy side effects: Expectations, doctor-patient communication and impact on quality of life - An Italian survey. Eur J Cancer Care (Engl). 2017;26(2).
12. Jacobs JM, Pensak NA, Sporn NJ, MacDonald JJ, Lennes IT, Safren SA, et al. Treatment Satisfaction and Adherence to Oral Chemotherapy in Patients With Cancer. J Oncol Pract. 2017;13(5):e474-e85.
13. Krishnasamy M, Hyatt A, Chung H, Gough K, Fitch M. Refocusing cancer supportive care: a framework for integrated cancer care. Support Care Cancer. 2022;31(1):14.
14. Nekhlyudov L. Introduction and Overview. In: Nekhlyudov L, Goel MS, Lin JJ, Overholser L, Peairs KS, editors. Caring for Patients Across the Cancer Care Continuum: Essentials for Primary Care. Cham: Springer International Publishing; 2019. p. 1-5.
15. Shelburne N, Simonds NI, Jensen RE, Brown J. Cancer-related emergency and urgent care: expanding the research agenda. Emergency Cancer Care. 2022;1(1):4.
16. Heins MJ, Lorenzi MF, Korevaar JC, McBride ML. Non-oncology physician visits after diagnosis of cancer in children. BMC Family Practice. 2016;17(1):60.

For peer review only - http://bmjopen.bmj.com/site/about/guidelines.xhtml

17. Ianovici C, Purcărea VL, Gheorghe IR, Blidaru A. The complexity of physician-patient communication and its impact in non-medical fields. A surgical oncology approach. J Med Life. 2023;16(4):631-4.
18. Marron JM, Cronin AM, Kang TI, Mack JW. Intended and unintended consequences: Ethics, communication, and prognostic disclosure in pediatric oncology. Cancer. 2018;124(6):1232-41.
19. Steven B, Lange L, Schulz H, Bleich C. Views of psycho-oncologists, physicians, and nurses on cancer care-A qualitative study. PLoS One. 2019;14(1):e0210325.
20. Collet R, Major M, van Egmond M, van der Leeden M, Maccow R, Eskes A, et al. Experiences of interaction between people with cancer and their healthcare professionals: A systematic review and meta-synthesis of qualitative studies. Eur J Oncol Nurs. 2022;60:102198.
21. Moore PM, Rivera S, Bravo-Soto GA, Olivares C, Lawrie TA. Communication skills training for healthcare professionals working with people who have cancer. Cochrane Database Syst Rev. 2018;7(7):Cd003751.
22. Gilligan T, Coyle N, Frankel RM, Berry DL, Bohlke K, Epstein RM, et al. Patient-Clinician Communication: American Society of Clinical Oncology Consensus Guideline. J Clin Oncol. 2017;35(31):3618-32.
23. Lounsbury DW, Nichols S, Asuzu C, Odiyo P, Alis A, Qadir M, et al. Communicating bad news to patients and families in African oncology settings. Psychooncology. 2023;32(1):47-57.
24. Henry M, Nichols S, Hwang JM, Nichols SD, Odiyo P, Watson M, et al. Barriers to communicating a cancer diagnosis to patients in a low- to middle-income context. Journal of Psychosocial Oncology Research and Practice. 2021;3(2):e049.
25. Horlait M, Van Belle S, Leys M. The need for adequate communication training programs for palliative care in multidisciplinary teams in oncology settings. International Journal of Integrated Care. 2016.
26. Horlait M, Chambaere K, Pardon K, Deliens L, Van Belle S. What are the barriers faced by medical oncologists in initiating discussion of palliative care? A qualitative study in Flanders, Belgium. Supportive Care in Cancer. 2016;24(9):3873-81.
27. He M, Zhu X, Dong Y, Zhang S, Fang S, Wang W, et al. Exploring the role of communication in missed nursing care: A systematic review. J Adv Nurs. 2022;78(12):4019-33.
28. Kwame A, Petrucka PM. A literature-based study of patient-centered care and communication in nurse-patient interactions: barriers, facilitators, and the way forward. BMC Nurs. 2021;20(1):158.
29. Garcia-Vivar C, Elizondo N, Ambrosio L. Primary Care Nursing Is Essential to Fully Implement Survivorship Care Plans for Long-term Cancer Survivors and Their Families. Cancer Nursing. 2019;42(3):177-8.
30. Peters M, Godfrey C, McInerney P, Munn Z, Tricco A, Khalil H. Chapter 11: Scoping Reviews (2020 version). In: Aromataris E, Munn Z, editors. JBI Manual for Evidence Synthesis: JBI; 2020.
31. Arksey H, O'Malley L. Scoping studies: towards a methodological framework. International Journal of Social Research Methodology. 2005;8(1):19-32.
32. Levac D, Colquhoun H, O'Brien KK. Scoping studies: advancing the methodology. Implementation Science. 2010;5(1):69.
33. Tricco AC, Lillie E, Zarin W, O'Brien KK, Colquhoun H, Levac D, et al. PRISMA Extension for Scoping Reviews (PRISMA-ScR): Checklist and Explanation. Ann Intern Med. 2018;169(7):467-73.

For peer review only - http://bmjopen.bmj.com/site/about/guidelines.xhtml

34. Lockwood C, dos Santos KB, Pap R. Practical Guidance for Knowledge Synthesis: Scoping Review Methods. Asian Nursing Research. 2019;13(5):287-94.
35. Aromataris E, Riitano D. Systematic Reviews: Constructing a Search Strategy and Searching for Evidence. AJN The American Journal of Nursing. 2014;114(5).
36. Thorn H, Uhrenfeldt L. Experiences of non-specialist nurses caring for patients and their significant others undergoing transitions during palliative end-of-life cancer care: a systematic review. JBI Database System Rev Implement Rep. 2017;15(6):1711-46.
37. Graetz DE, Garza M, Rodriguez-Galindo C, Mack JW. Pediatric cancer communication in low- and middle-income countries: A scoping review. Cancer. 2020;126(23):5030-9.
38. EBSCO Information Services. EBSCOhost Research Platform 2023 [Available from: https://www.ebsco.com/products/ebscohost-research-platform.
39. Clarivate. EndNote 20 2023 [
40. Veritas Health Innovation. Covidence systematic review software, Melbourne, Australia.2023 [Available from: Available at www.covidence.org.
41. Peterson J, Pearce PF, Ferguson LA, Langford CA. Understanding scoping reviews: Definition, purpose, and process. J Am Assoc Nurse Pract. 2017;29(1):12-6.
42. Pollock D, Davies EL, Peters MDJ, Tricco AC, Alexander L, McInerney P, et al. Undertaking a scoping review: A practical guide for nursing and midwifery students, clinicians, researchers, and academics. Journal of Advanced Nursing. 2021;77(4):2102-13.
43. Munn Z, Stone JC, Aromataris E, Klugar M, Sears K, Leonardi-Bee J, et al. Assessing the risk of bias of quantitative analytical studies: introducing the vision for critical appraisal within JBI systematic reviews. JBI Evidence Synthesis. 2023;21(3):467-71.

For peer review only

For peer review only - http://bmjopen.bmj.com/site/about/guidelines.xhtml

AUTHORS' CONTRIBUTION

CK conceptualised the study, developed the methods, developed the preliminary search strategy, wrote the first draft, critically reviewed the draft manuscript and approved the final draft for submission.

LH conceptualised the study, developed the methods, developed the preliminary search strategy, critically reviewed the draft manuscript and approved the final draft for submission.

JM conceptualised the study, critically reviewed the draft manuscript and approved the final draft for submission.

NC provided critical inputs to methods and editing of this protocol, critically reviewed the draft manuscript and approved the final draft for submission.

COMPETING INTERESTS

Non declared.

FUNDING

This research received no specific grant from any funding agency in the public, commercial or not-for-profit sector.

For peer review only

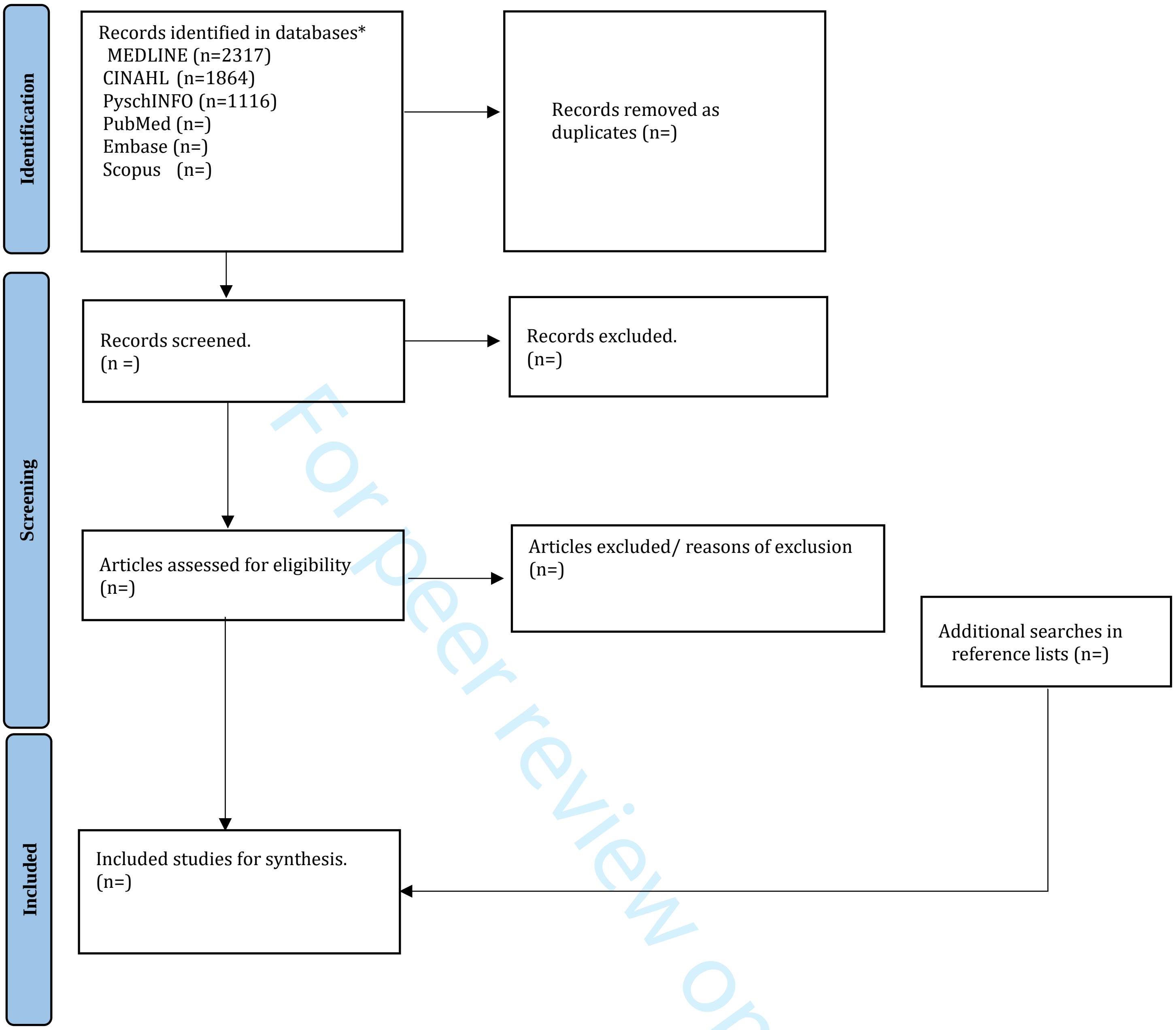

For peer review only

Appendix 1 Scoping search results

On 24 October 2022, a scoping search was conducted to check if this scoping review (or any review) is in process or has been conducted in the past. JBI systematic review register, Prospero, Cochrane register, and PubMed was searched using the simple search term "cancer communication". The table below summarises the findings:

Database	Summary
Prospero	The search retrieved nine records.<br>No registered review of the proposed topic.
JBI systematic review register	Only three articles were retrieved. No article included cancer communication.
PubMed	After filtering by meta-analysis, reviews, and systematic reviews, the search term "cancer communication*" yielded 136 studies. No review was similar to this scoping review objectives.
Cochrane	The search retrieved 35 records. No review was similar to this scoping review objectives.

For peer review only

Appendix 2 Initial data extraction

MEDLINE

1	Describing the population of this study	Results
Participants	(MH "Nursing Staff+") OR (MH "Nurses+") OR (MH "Nursing+") OR (MH "Nursing Care+") OR (MH "Nurse Practitioners+") OR (MH "Nursing Staff, Hospital+") OR "nursing staff" OR nurs* OR "nursing care" OR "nurse practitioner*" OR "hospital nurse*" OR "non-speciali#ed nurse*"	633,365
2	Describing the concepts –Cancers	
Cancers	(MH "Neoplasms+") OR neoplasm* OR neoplasia Or tum#r* OR "Malignant neoplasm*" OR Malignan* OR cancer* OR "cancer patient*"	4,718,229
3	Describing the concepts-communication and experiences	
Communication and communication experience	(MH "Communication+") OR (MH "Communication Methods, Total") OR (MH "Disclosure+") OR communication OR "information exchange" OR "communication pattern*" OR "communication strategy*" OR "communication method*" OR	3,060,614

For peer review only

	"communication technique*" OR disclosure OR interaction* OR experience*	
4	Combining all concepts	
	1 AND 2 AND 3	9,478
5	Limiter 1: Date between 2012 and June 2022	4,962
6	Limiter 2: adult 19 years and above	2,393
7	Limiter 3: English publication	2,317

CINAHL

1	Describing the population of this study	Results
Participants	(MH "Nurses+") OR (MH "Nursing Care+") OR “nursing staff” OR nurse* OR "nursing care" OR "nurse practitioner*" OR "hospital nurse*" OR "non-speciali#ed nurse*."	741,262
2	Describing the concepts –Cancers	
Cancers	(MH "Neoplasms+") OR (MH "Cancer Patients") OR neoplasm* OR neoplasia Or tum#r* OR “Malignant neoplasm*” OR	795,776

For peer review only

	Malignan* OR cancer* OR "cancer patients"	
3	Describing the concepts-communication and experiences	
Communication and communication experience	(MH "Communication+") OR (MH "Communication Methods, Total") OR (MH "Disclosure+") OR communication OR "information exchange" OR "communication pattern*" OR "communication strategy*" OR "communication method*" OR "communication technique*" OR disclosure OR interaction* OR experience*	895,801
4	Combining all concepts	
	1 AND 2 AND 3	9,442
5	Limiter 1: Date between 2012 and June 2022	5,188
6	Limiter 2: all adults (18 and above)	1,940
7	Limiter 3: English publication	1,864

For peer review only

PsycINFO

1	Describing the population of this study	Results
Participants	MM "Nursing" OR DE "Nurses" OR DE "Psychiatric Nurses" OR DE "Public Health Service Nurses" OR DE "School Nurses" OR “nursing staff” OR nurs* OR “nursing care” OR “nurse practitioner*” OR “hospital nurse*” OR "non-speciali#ed nurse*"	111,885
2	Describing the concepts –Cancers	
Cancers	DE "Neoplasms" OR DE "Benign Neoplasms" OR DE "Breast Neoplasms" OR DE "Endocrine Neoplasms" OR DE "Leukemias" OR DE "Melanoma" OR DE "Metastasis" OR DE "Nervous System Neoplasms" OR DE "Terminal Cancer" OR neoplasia OR tum#r* OR “Malignant neoplasm*” OR Malignan* OR cancer* OR cancer patient*	90,760

For peer review only

3	Describing the concepts-communication and experiences	
Communication and communication experience	DE "Communication" OR DE "Augmentative Communication" OR DE "Communication Barriers" OR DE "Electronic Communication" OR DE "Interpersonal Communication" OR DE "Messages" OR DE "Nonverbal Communication" OR DE "Persuasive Communication" OR DE "Privileged Communication" OR DE "Scientific Communication" OR DE "Social Communication" OR DE "Verbal Communication" OR communication OR “information exchange” OR “communication pattern*” OR “communication strategy*" OR "communication method*" OR "communication technique*" OR disclosure OR interaction* OR experience*	1,183,559
4	Combining all concepts	
	1 AND 2 AND 3	2,450

For peer review only

5	Limiter 1: Date between 2012 and June 2022	1,413
6	Limiter 2: adult 18 years and above	1,125
7	Limiter 3: English publication	1,116

For peer review only

1

PRISMA-P 2015 Checklist

This checklist has been adapted for use with protocol submissions to *Systematic Reviews* from Table 3 in Moher D et al: Preferred reporting items for systematic review and meta-analysis protocols (PRISMA-P) 2015 statement. *Systematic Reviews* 2015 **4**:1

Section/topic	#	Checklist item	Information reported Yes	Information reported No	Line number(s)
**ADMINISTRATIVE INFORMATION**					
**Title**					
Identification	1a	Identify the report as a protocol of a systematic review	☒	☐	Check title
Update	1b	If the protocol is for an update of a previous systematic review, identify as such	☐	☐	NA
**Registration**	2	If registered, provide the name of the registry (e.g., PROSPERO) and registration number in the Abstract	☐	☒	
**Authors**					
Contact	3a	Provide name, institutional affiliation, and e-mail address of all protocol authors; provide physical mailing address of corresponding author	☒	☐	
Contributions	3b	Describe contributions of protocol authors and identify the guarantor of the review	☒	☐	Check authors contribution
**Amendments**	4	If the protocol represents an amendment of a previously completed or published protocol, identify as such and list changes; otherwise, state plan for documenting important protocol amendments	☐	☐	NA
**Support**					
Sources	5a	Indicate sources of financial or other support for the review	☒	☐	Check funding statement
Sponsor	5b	Provide name for the review funder and/or sponsor	☐	☐	NA
Role of sponsor/funder	5c	Describe roles of funder(s), sponsor(s), and/or institution(s), if any, in developing the protocol	☐	☐	NA
**INTRODUCTION**					
**Rationale**	6	Describe the rationale for the review in the context of what is already known	☒	☐	Introduction section
**Objectives**	7	Provide an explicit statement of the question(s) the review will address with reference to participants, interventions, comparators, and outcomes (PICO)	☒	☐	Aims subsection

BioMed Central The Open Access Publisher

Section/topic	#	Checklist item	Information reported		Line number(s)
			Yes	No	
**METHODS**					
**Eligibility criteria**	8	Specify the study characteristics (e.g., PICO, study design, setting, time frame) and report characteristics (e.g., years considered, language, publication status) to be used as criteria for eligibility for the review	☒	☐	Check inclusion criteria and table 1
**Information sources**	9	Describe all intended information sources (e.g., electronic databases, contact with study authors, trial registers, or other grey literature sources) with planned dates of coverage	☒	☐	Check sources of evidence and search strategy and information sources section
**Search strategy**	10	Present draft of search strategy to be used for at least one electronic database, including planned limits, such that it could be repeated	☒	☐	Check search strategy and information sources and table 2
STUDY RECORDS					
Data management	11a	Describe the mechanism(s) that will be used to manage records and data throughout the review	☒	☐	Check data extraction and assessment of the methodological quality section.
Selection process	11b	State the process that will be used for selecting studies (e.g., two independent reviewers) through each phase of the review (i.e., screening, eligibility, and inclusion in meta-analysis)	☒	☐	Check study selection
Data collection process	11c	Describe planned method of extracting data from reports (e.g., piloting forms, done independently, in duplicate), any processes for obtaining and confirming data from investigators	☒	☐	Check presentation of results section
**Data items**	12	List and define all variables for which data will be sought (e.g., PICO items, funding sources), any pre-planned data assumptions and simplifications	☒	☐	Check Data extraction and assessment of methodological quality section
**Outcomes and prioritization**	13	List and define all outcomes for which data will be sought, including prioritization of main and additional outcomes, with rationale	☒	☐	Check Data extraction and assessment of

BioMed Central The Open Access Publisher

Section/topic	#	Checklist item	Information reported Yes	No	Line number(s)
					methodological quality section
**Risk of bias in individual studies**	14	Describe anticipated methods for assessing risk of bias of individual studies, including whether this will be done at the outcome or study level, or both; state how this information will be used in data synthesis	☒	☐	Check Data extraction and assessment of methodological quality section
DATA					
**Synthesis**	15a	Describe criteria under which study data will be quantitatively synthesized	☐	☐	NA
	15b	If data are appropriate for quantitative synthesis, describe planned summary measures, methods of handling data, and methods of combining data from studies, including any planned exploration of consistency (e.g., I^2, Kendall's tau)	☐	☐	NA
	15c	Describe any proposed additional analyses (e.g., sensitivity or subgroup analyses, meta-regression)	☐	☐	NA
	15d	If quantitative synthesis is not appropriate, describe the type of summary planned	☒	☐	Check Data extraction and assessment of methodological quality section
**Meta-bias(es)**	16	Specify any planned assessment of meta-bias(es) (e.g., publication bias across studies, selective reporting within studies)	☐	☐	NA
**Confidence in cumulative evidence**	17	Describe how the strength of the body of evidence will be assessed (e.g., GRADE)	☐	☐	NA

For peer review only

BioMed Central The Open Access Publisher

Communication to Adult Patients Undergoing Cancer Care by Non-Specialist Nurses: a Scoping Review Protocol

Journal:	*BMJ Open*
Manuscript ID	bmjopen-2023-081326.R2
Article Type:	Protocol
Date Submitted by the Author:	01-Mar-2024
Complete List of Authors:	Kachimanga, Chiyembekezo; University of Stirling<br>McGlashan, Jennifer; University of Stirling<br>Cunningham, Nicola; University of Stirling,<br>Hoyle, Louise; University of Stirling
**Primary Subject Heading**:	Nursing
Secondary Subject Heading:	Oncology, Communication
Keywords:	ONCOLOGY, Systematic Review, Nursing Care, Nurses

Communication to Adult Patients Undergoing Cancer Care by Non-Specialist Nurses: a Scoping Review Protocol

Target journal: BMJ Open

Authors

Chiyembekezo Kachimanga[1], Jennifer McGlashan [1], Nicola Cunningham [1], Louise Hoyle[1]

Affiliation

1. University of Stirling

*Corresponding author

Chiyembekezo Kachimanga

Email: chk00095@students.stir.ac.uk

Word count: 2747

For peer review only

ABSTRACT

Introduction: Little is known regarding how non-specialist nurses communicate with patients living with cancer when the patients are receiving care outside of their cancer units/teams. This scoping review aims to identify, examine and report on the currently available evidence about communication by non-specialist nurses when caring for adults living with cancer outside of their cancer care unit/teams.

Methods and analysis: A scoping review following the JBI methodology for scoping reviews will be conducted. We will search for empirical studies that meet the inclusion criteria in six databases (MEDLINE, PubMed, CINAHL, Embase, Scopus, and PsycINFO). Hand-searching in references of included articles will be performed to find additional articles. The population of interest will be non-specialist nurses. Three concepts will be explored, namely 1) all adult patients living with cancer, 2) a focus on three stages of the cancer continuum of care (cancer diagnosis, treatment, and survivorship), and 3) a focus on communication between non-specialist nurses and patients living with cancer. We will include studies describing all healthcare settings outside patients' specialised cancer units or oncology teams. After article selection, two reviewers will independently screen titles and abstracts and perform a full-text article review, risk of bias assessments and data extraction. A third reviewer will resolve all disagreements. A narrative summary will provide an overview of how the results relate to the research aims and questions. The included articles will be limited to English and published between 2012 and 2023

Ethics and dissemination: No ethical approval is required since we will use publicly available empirical research sources**.** This review will provide current research on communication by non-specialist nurses with patients with a cancer diagnosis outside

For peer review only - http://bmjopen.bmj.com/site/about/guidelines.xhtml

of an oncology setting, evidence that will support effective communication. As such, we aim to disseminate the findings in academic conferences and peer-reviewed journals.

Protocol registration: Open Science Framework registry (https://osf.io/3pmt9)

Keywords: cancer, neoplasms, communication, scoping reviews, non-specialist nurses

Strengths and Limitations of this study

1. This will be the first review that provides a comprehensive summary of communication by non-specialist nurses with patients with a cancer diagnosis outside of an oncology setting.
2. The scoping review will be systematic and transparent as it will be guided by JBI methodology for scoping reviews and will be reported using the checklist for preferred reporting for systematic reviews and meta-analyses extension for scoping reviews (PRISMA -ScR).
3. The review will only focus on empirical studies published in academic journals and will exclude articles published in grey literature.
4. The review will be limited to studies published in English between 2012 and 2023.

For peer review only - http://bmjopen.bmj.com/site/about/guidelines.xhtml

INTRODUCTION

Cancer is one of the most commonly diagnosed non-communicable diseases and the leading cause of death worldwide(1). In 2020 alone, over 20 million people were diagnosed with cancer, and nearly 17% of all deaths were caused by cancer (2). Cancers affect all regions of the world, and the incidence of cancers will increase by up to 50% by 2040(3).

The higher morbidity and mortality of cancer require appropriate care at all health system levels. Cancer care is provided by a multidisciplinary team from the community to tertiary care facilities. Among the essential healthcare providers are the nurses, the most numerous healthcare providers working with people diagnosed with cancer(4). Whether generalists or specialists, nurses play essential roles in managing patients diagnosed with cancer through screening, treatment, survivorship, or end-of-life care(5). Nurse-led cancer interventions, which include direct patient care, education, counselling and care coordination, improve high-quality patient-centred cancer care(6). Patients with cancer diagnoses need care from a well-trained, updated, and skilled nursing workforce(7).

Patients living with cancer require effective communication from their healthcare providers. Effective communication means providing all necessary information to allow a good understanding of their cancer diagnosis and managing emotional responses stemming from an awareness of the diagnosis (8). Effective communication also helps patients with a cancer diagnosis make informed care decisions, fosters strong relationships between patients and their providers (9), improves clinical outcomes and improves patients' care experience (10). Furthermore, effective communication

For peer review only - http://bmjopen.bmj.com/site/about/guidelines.xhtml

enhances patient satisfaction and quality of life, reduces providers' stress and burnout, improves adherence to cancer care, and eases caretaker burden(9, 11, 12).

Communication with patients by healthcare providers is bi-directional and provided throughout the cancer care continuum(13). The bi-directional nature entails that healthcare providers provide information that allows patients to make decisions based on their understanding of the disease, and patients are empowered to make decisions that will ultimately improve their care(8). The cancer continuum depicts the steps involved in cancer care and control and includes 1) prevention and risk reduction, 2) screening, 3) cancer diagnosis, 4) treatment, 4) survivorship, and 5) end-of-life care (14). Throughout this cancer continuum, communication is provided by all healthcare providers involved in patient care. Communication with patients with a cancer diagnosis does not only happen when patients receive care from their oncology teams but also during any encounter with health care providers, including care outside their oncology teams, emergency settings and non-cancer related clinical visits (15, 16). Regardless of the setting of clinical encounters, communication should be continuous and reinforced over time.

Effective communication is widely recognised as one of the dimensions of quality in cancer care (17). However, despite this recognition, effective communication remains one of the most common unmet needs for patients with cancer (9). Effective communication goes beyond providing understandable information; it involves communicating in a patient-centred approach- a process where communication is provided sensitively, with respect to patient's autonomy and involving patients in the decision-making process (18, 19).

For peer review only

For peer review only - http://bmjopen.bmj.com/site/about/guidelines.xhtml

Communication in cancer care is a learned clinical skill and goes beyond medical interviews due to the complexity and life-threatening issues commonly discussed with patients living with cancer (20). As such, for oncologists and other healthcare providers working in cancer units, rigorous communication training should be provided as part of their initial and routine training(21, 22). However, even if communication training is in place, evidence suggests that trained healthcare providers need help with effective communication. For example, a study in cancer care settings in Africa found that only 40% of nurses and 20% of physicians had formal communications training (23). Similar studies in Kenya and Belgium have also shown that lack of communication training is a major challenge in cancer care(24-26). In these studies, emphasis was placed on expanding and improving communication training during pre-service training, providing further guidance and mentorship during cancer care coordination and adapting communication to different contexts and/or cultural backgrounds.

Patients with a cancer diagnosis may require care outside their usual care teams, and nurses play a more prominent role in this context. Nurses, including non-specialised nurses, are usually the first-line workers, they spend the most of their time with clients and interface with cancer patients during outpatient and in-patient services (27, 28). Non-specialist nurses also have essential roles in cancer care which include: direct nursing care, patient navigation, educating other healthcare providers, and care coordination (29).

To ensure good outcomes, non-specialist nurses must be equipped with practical communication skills when caring for an individual diagnosed with cancer. Little is known regarding how non-specialist nurses communicate with patients living with cancer when receiving care outside their cancer units/teams. This scoping review aims

For peer review only - http://bmjopen.bmj.com/site/about/guidelines.xhtml

to understand non-specialist nurses' roles and skills in effectively communicating with adult patients living with cancer.

Aims

The main objective of this scoping review will be to identify the roles and skills of non-specialist nurses in providing effective communication to adult patients with a diagnosis of cancer receiving any health services outside of their cancer care units. An initial scoping search was conducted in four databases (Prospero, PubMed, JBI systematic review register and Cochrane database) to check if any review is in progress or has been published on this scoping review objective. No review similar to this topic is being conducted or has been published (Appendix 1). This scoping review will answer four main questions:

1. What are the roles of non-specialist nurses in providing effective communication to adult patients with a diagnosis of cancer attending any health service outside of their cancer units?
2. When do non-specialist nurses communicate with adult patients with cancer attending any health services outside of cancer units?
3. How do non-specialist nurses communicate with adult patients with cancer attending any health services outside of cancer units?
4. What communication skills do they have to effectively communicate to adult patients with cancer attending any health services outside of cancer units?

METHODS AND ANALYSIS

To identify, examine and report on the currently available evidence about communication by non-specialist nurses when caring for an individual with a diagnosis of cancer, the most appropriate approach to gathering and synthesising evidence will be

For peer review only - http://bmjopen.bmj.com/site/about/guidelines.xhtml

a scoping review. This review will be guided by the JBI methodology for scoping reviews (30). The JBI methodology for scoping reviews was advanced and refined from two previous scoping review guidance from Arksey and O'Malley(31) and Levac et al.(32). Furthermore, will use the checklist for preferred reporting for systematic reviews and meta-analyses extension for scoping reviews (PRISMA -ScR)(33). We registered the protocol in Open Science Framework registry (https://osf.io/3pmt9)

Inclusion criteria

To define the scope and inclusion criteria of the review, we have used the population, concepts, and context (PCC) framework (30, 34). The population of interest will be non-specialist nurses. Three concepts will be explored, namely 1) All adult patients (18 years and above) living with cancer, 2) a focus on three stages of the cancer continuum of care (cancer diagnosis, treatment, and survivorship), 3) a focus on communication between non-specialist nurses and patients living with cancer. In context, we will include studies conducted in any geographical location describing all healthcare settings outside patients' specialised cancer units or oncology teams. Table 1 details the inclusion and exclusion criteria.

For peer review only - http://bmjopen.bmj.com/site/about/guidelines.xhtml

Table 1 Inclusion and exclusion criteria

Inclusion criteria	Exclusion criteria
1) Studies reporting on non-specialist nurses. Non-specialist nurses are not specifically trained to provide expert advice and care for cancer patients and do not have formal certification in cancer care.<br>2) Patients living with cancer aged 18 years and above.<br>3) Patients living with cancer in any of the three stages of the cancer continuum of care (diagnosis, treatment and survivorship)<br>4) Studies reporting on communication between non-specialist nurses and patients diagnosed with cancer. We will explore the following concepts:<br>a. Experiences and roles of non-specialist nurses in communicating with cancer patients<br>b. Context where communication between non-specialist nurses and cancer patients occurs	1) Studies reporting on specialist nurses, student nurses or other health care workers.<br>2) Studies reporting on cancer screening, end-of-life care or palliative care stage of cancer continuum of care<br>3) Studies not reporting on communication.<br>4) Studies reporting on patients that cannot communicate or have limited capacity to communicate, e.g. Dementia and Alzheimer's disease.

For peer review only - http://bmjopen.bmj.com/site/about/guidelines.xhtml

c. How communication (verbal and non-verbal) occurs between non-specialist nurses and patients with cancer<br>d. Training and skills in communicating with cancer patients by non-specialist nurses<br>5) Communication occurs when cancer patients access services outside of the cancer unit /care teams<br>6) Studies published in the English language<br>7) Studies conducted between 2012 and 2023<br>8) Qualitative, quantitative, or mixed-methods study designs	

Sources of evidence

The review will consider empirical studies from peer-reviewed articles that used quantitative, qualitative, and mixed-method designs. All designs within these types of studies will be included. All other study types, including review studies, commentaries, policy-related documents, and conference proceedings, will be excluded. We will also hand-search references of included articles to identify additional articles. As the authors are proficient in the English language only, we will include studies published in the English language only. We also plan to review recent articles on communication; hence,

For peer review only

we will restrict studies published in the last decade (Between January 2012 and December 2023).

Search strategy and information sources

This review aims to identify published empirical studies. The search strategy will follow a three-step process as recommended by the JBI methodology for scoping reviews(35). In step 1, an initial limited search was undertaken in October 2022. The following concepts were used as the initial starting point: "non-specialist nurse" AND Cancer AND communication. Each concept term was expanded by looking at synonyms and identifying common terms used in the literature (21, 36, 37). Using the EBSCOhost platform for Medline, CINAHL and PsycINFO databases, appropriate medical subject headings and free text for each concept were developed (38). The 'expand' option to expand some of the subject headings was used as appropriate. CK designed the initial search terms, which LH validated. Table 2 provides an overview of the sample search terms used at this initial point, and Appendix 2 provides the results of this limited search. The titles, abstract and index terms will be analysed from this initial search to inform the final search strategy.

For peer review only - http://bmjopen.bmj.com/site/about/guidelines.xhtml

1 *Table 2 Sample Search Terms*

2

1	Describing the population of this study
Participants	(MH "Nursing Staff+") OR (MH "Nurses+") OR (MH "Nursing+") OR (MH "Nursing Care+") OR (MH "Nurse Practitioners+") OR (MH "Nursing Staff, Hospital+") OR "nursing staff" OR nurs* OR "nursing care" OR "nurse practitioner*" OR "hospital nurse*" OR "non-speciali#ed nurse*"
2	Describing the concepts –Cancers
Cancers	(MH "Neoplasms+") OR neoplasm* OR neoplasia Or tum#r* OR "Malignant neoplasm*" OR Malignan* OR cancer* OR "cancer patient*"
3	Describing the concepts-communication and experiences
Communication and communication experience	(MH "Communication+") OR (MH "Communication Methods, Total") OR (MH "Disclosure+") OR communication OR "information exchange" OR "communication pattern*" OR "communication strategy*" OR "communication method*" OR "communication technique*" OR disclosure OR interaction* OR experience*
4	Combining all concepts
	1 AND 2 AND 3
5	Limiter 1: Date between 2012 and June 2022
6	Limiter 2: adult 18 years and above

For peer review only - http://bmjopen.bmj.com/site/about/guidelines.xhtml

7	Limiter 3: English publication

In step 2, we will perform another search using the developed search strategy from the previous step. A comprehensive search in the MEDLINE, PubMed, CINAHL, Embase, Scopus, and PsycINFO databases will be performed. In step 3, additional studies will be supplemented by hand-searching the references of included sources for additional citations.

Study selection

After a comprehensive data search in the selected databases, we will export all articles to EndNote software version 20, where all duplicates will be removed (39). Afterwards, all articles will be exported to Covidence software for article screening, full-text review and data extraction (40). Two reviewers will independently review the title and abstracts to identify articles eligible for full-text review, conduct a full-text review to identify articles eligible for data extraction and perform data extraction based on a piloted data extraction tool. A third reviewer will resolve all disagreements during the title and abstract screening, full-text screening, and data extraction. The full text of retrieved sources that do not meet the inclusion criteria will be excluded, but a note will be made for the reason for exclusion. The searching, screening and selection will be documented using a PRISMA-ScR flow diagram (figure 1). The flow diagram will be updated once the review has been completed.

Figure 1 PRISMA flow diagram

NB * The search results will be update by the end of the review

For peer review only

Data extraction and assessment of methodological quality

We will document the data based on a standardised data collection tool. Before data collection, the tool will be piloted on a selection of articles eligible for the title and abstract screening (30). Two reviewers will independently perform the extraction, followed by a discussion to resolve differences and create the final data collection tool (see table 3 for sample data collection tool). We plan to extract the following data based on the eligibility criteria:

1) Details of the article: author, year, and study design
2) Context: country of the study and health care setting
3) Cancer care continuum
4) Types and roles played by non-specialist nurses in cancer care
5) Timing, frequency and contexts of cancer communication
6) Communication methods used
7) Communication training and on-going skills (pre-service and in-service skills) acquired by non-specialist nurses.

Despite pre-specifying the type of data we will collect, we will adjust the data that will be collected during data extraction if necessary. All modifications to the data collection will be described during the final report of the scoping review.

For peer review only - http://bmjopen.bmj.com/site/about/guidelines.xhtml

Table 3 Sample data collection tool

Author, Year	Study design	Participants	Context	Cancer continuum of care	Type/roles of non-specialist nurses	Timing of cancer communication	Communication methods	Communication training/skills

Although it is not mandatory to perform risk of bias assessment in scoping reviews, we will include risk of bias assessment in this scoping review (41, 42). Risk of bias assessment is an appraisal of the methodological quality of the included studies in-order to inform the synthesis of the collected data(43). In this review, we will use risk of bias assessment to identify studies that should be included with the purpose of including studies with low risk of biases. Appropriate tools will be identified using JBI critical appraisal tools (https://jbi.global/critical-appraisal-tools). Two reviewers will independently perform a risk of bias assessment, and a third reviewer will resolve all disagreements.

Presentation of results and expert consultation.

We plan to map the evidence based on PCC using both quantitative and qualitative study designs. For quantitative designs, a narrative summary will be written to provide an overview of how the results relate to the research aims and questions. For qualitative designs, descriptive content analysis will be used. Tables and figures will be used to provide an overview of extracted data relevant to the research questions. We plan to

For peer review

continue the comprehensive literature search in April 2024 and finalise the review by March 2025.

During the comprehensive literature search and data extraction we will seek additional support from subject-specific experts. We will seek an expert information specialist to validate the final search strategy. We will also seek support from a cancer expert to validate the findings prior to final dissemination.

Patient and public involvement

There is no patient or public involvement in this study.

DISCUSSION

To our knowledge, this will be the first review that looks at communication by non-specialist nurses with patients with a cancer diagnosis outside of an oncology setting. Patients living with cancer require comprehensive information regarding their diagnosis, which is often conveyed during provider-patient communication. While effective communication brings hope, peace of mind and trust in healthcare providers, ineffective communication leads to sub-optimal cancer care, increased patient and caregiver distress and lack of trust in healthcare providers (20).

Understanding the communication between non-specialised nurses and patients with a cancer diagnosis has implications for practice, policy and research. From a practical point of view, this review will highlight gaps in cancer communication, which may inform areas where clinical guidelines and training need to be included. The results may also influence policymakers by introducing narrative evidence on best practices or gaps in cancer communication by non-specialised nurses. Finally, the study will help identify gaps in the literature on cancer communication. Based on these gaps, further studies,

For peer review only - http://bmjopen.bmj.com/site/about/guidelines.xhtml

including systematic reviews, may be developed to understand specific aspects of cancer communication.

ETHICS AND DISSEMINATION

Ethical approval will not be needed as we will use publicly available published empirical studies. We plan to disseminate the results in nursing specific conferences and other academic conferences. The final report will be published in peer-reviewed academic journal.

For peer review only

For peer review only - http://bmjopen.bmj.com/site/about/guidelines.xhtml

REFERENCES

1. World Health Organization. Cancer 2022 [updated 3 february 2022. Available from: https://www.who.int/news-room/fact-sheets/detail/cancer.
2. Ferlay J, Ervik M, Lam F, Colombet M, Mery L, Piñeros M, et al. Global Cancer Observatory: Cancer Today Lyon, France: International Agency for Research on Cancer; 2020 [Available from: https://gco.iarc.fr/today.
3. Sung H, Ferlay J, Siegel RL, Laversanne M, Soerjomataram I, Jemal A, et al. Global Cancer Statistics 2020: GLOBOCAN Estimates of Incidence and Mortality Worldwide for 36 Cancers in 185 Countries. CA Cancer J Clin. 2021;71(3):209-49.
4. Challinor JM, Alqudimat MR, Teixeira TOA, Oldenmenger WH. Oncology nursing workforce: challenges, solutions, and future strategies. The Lancet Oncology. 2020;21(12):e564-e74.
5. Dowling M, Efstathiou N, Drury A, Semple C, Fernández-Ortega P, Brochstedt Dieperink K, et al. Cancer nursing research priorities: A rapid review. Eur J Oncol Nurs. 2023;63:102272.
6. Charalambous A, Wells M, Campbell P, Torrens C, Östlund U, Oldenmenger W, et al. A scoping review of trials of interventions led or delivered by cancer nurses. International Journal of Nursing Studies. 2018;86:36-43.
7. Young AM, Charalambous A, Owen RI, Njodzeka B, Oldenmenger WH, Alqudimat MR, et al. Essential oncology nursing care along the cancer continuum. Lancet Oncol. 2020;21(12):e555-e63.
8. National Cancer Institute. Communication in Cancer Care 2022 [Available from: https://www.cancer.gov/about-cancer/coping/adjusting-to-cancer/communication-hp-pdq.
9. Li J, Luo X, Cao Q, Lin Y, Xu Y, Li Q. Communication Needs of Cancer Patients and/or Caregivers: A Critical Literature Review. Journal of Oncology. 2020;2020:7432849.
10. Lafata JE, Shay LA, Winship JM. Understanding the influences and impact of patient-clinician communication in cancer care. Health Expect. 2017;20(6):1385-92.
11. Lorusso D, Bria E, Costantini A, Di Maio M, Rosti G, Mancuso A. Patients' perception of chemotherapy side effects: Expectations, doctor-patient communication and impact on quality of life - An Italian survey. Eur J Cancer Care (Engl). 2017;26(2).
12. Jacobs JM, Pensak NA, Sporn NJ, MacDonald JJ, Lennes IT, Safren SA, et al. Treatment Satisfaction and Adherence to Oral Chemotherapy in Patients With Cancer. J Oncol Pract. 2017;13(5):e474-e85.
13. Krishnasamy M, Hyatt A, Chung H, Gough K, Fitch M. Refocusing cancer supportive care: a framework for integrated cancer care. Support Care Cancer. 2022;31(1):14.
14. Nekhlyudov L. Introduction and Overview. In: Nekhlyudov L, Goel MS, Lin JJ, Overholser L, Peairs KS, editors. Caring for Patients Across the Cancer Care Continuum: Essentials for Primary Care. Cham: Springer International Publishing; 2019. p. 1-5.
15. Shelburne N, Simonds NI, Jensen RE, Brown J. Cancer-related emergency and urgent care: expanding the research agenda. Emergency Cancer Care. 2022;1(1):4.
16. Heins MJ, Lorenzi MF, Korevaar JC, McBride ML. Non-oncology physician visits after diagnosis of cancer in children. BMC Family Practice. 2016;17(1):60.
17. Ianovici C, Purcărea VL, Gheorghe IR, Blidaru A. The complexity of physician-patient communication and its impact in non-medical fields. A surgical oncology approach. J Med Life. 2023;16(4):631-4.

For peer review only - http://bmjopen.bmj.com/site/about/guidelines.xhtml

18. Marron JM, Cronin AM, Kang TI, Mack JW. Intended and unintended consequences: Ethics, communication, and prognostic disclosure in pediatric oncology. Cancer. 2018;124(6):1232-41.
19. Steven B, Lange L, Schulz H, Bleich C. Views of psycho-oncologists, physicians, and nurses on cancer care-A qualitative study. PLoS One. 2019;14(1):e0210325.
20. Collet R, Major M, van Egmond M, van der Leeden M, Maccow R, Eskes A, et al. Experiences of interaction between people with cancer and their healthcare professionals: A systematic review and meta-synthesis of qualitative studies. Eur J Oncol Nurs. 2022;60:102198.
21. Moore PM, Rivera S, Bravo-Soto GA, Olivares C, Lawrie TA. Communication skills training for healthcare professionals working with people who have cancer. Cochrane Database Syst Rev. 2018;7(7):Cd003751.
22. Gilligan T, Coyle N, Frankel RM, Berry DL, Bohlke K, Epstein RM, et al. Patient-Clinician Communication: American Society of Clinical Oncology Consensus Guideline. J Clin Oncol. 2017;35(31):3618-32.
23. Lounsbury DW, Nichols S, Asuzu C, Odiyo P, Alis A, Qadir M, et al. Communicating bad news to patients and families in African oncology settings. Psychooncology. 2023;32(1):47-57.
24. Henry M, Nichols S, Hwang JM, Nichols SD, Odiyo P, Watson M, et al. Barriers to communicating a cancer diagnosis to patients in a low- to middle-income context. Journal of Psychosocial Oncology Research and Practice. 2021;3(2):e049.
25. Horlait M, Van Belle S, Leys M. The need for adequate communication training programs for palliative care in multidisciplinary teams in oncology settings. International Journal of Integrated Care. 2016.
26. Horlait M, Chambaere K, Pardon K, Deliens L, Van Belle S. What are the barriers faced by medical oncologists in initiating discussion of palliative care? A qualitative study in Flanders, Belgium. Supportive Care in Cancer. 2016;24(9):3873-81.
27. He M, Zhu X, Dong Y, Zhang S, Fang S, Wang W, et al. Exploring the role of communication in missed nursing care: A systematic review. J Adv Nurs. 2022;78(12):4019-33.
28. Kwame A, Petrucka PM. A literature-based study of patient-centered care and communication in nurse-patient interactions: barriers, facilitators, and the way forward. BMC Nurs. 2021;20(1):158.
29. Garcia-Vivar C, Elizondo N, Ambrosio L. Primary Care Nursing Is Essential to Fully Implement Survivorship Care Plans for Long-term Cancer Survivors and Their Families. Cancer Nursing. 2019;42(3):177-8.
30. Peters M, Godfrey C, McInerney P, Munn Z, Tricco A, Khalil H. Chapter 11: Scoping Reviews (2020 version). In: Aromataris E, Munn Z, editors. JBI Manual for Evidence Synthesis: JBI; 2020.
31. Arksey H, O'Malley L. Scoping studies: towards a methodological framework. International Journal of Social Research Methodology. 2005;8(1):19-32.
32. Levac D, Colquhoun H, O'Brien KK. Scoping studies: advancing the methodology. Implementation Science. 2010;5(1):69.
33. Tricco AC, Lillie E, Zarin W, O'Brien KK, Colquhoun H, Levac D, et al. PRISMA Extension for Scoping Reviews (PRISMA-ScR): Checklist and Explanation. Ann Intern Med. 2018;169(7):467-73.
34. Lockwood C, dos Santos KB, Pap R. Practical Guidance for Knowledge Synthesis: Scoping Review Methods. Asian Nursing Research. 2019;13(5):287-94.

For peer review only - http://bmjopen.bmj.com/site/about/guidelines.xhtml

35. Aromataris E, Riitano D. Systematic Reviews: Constructing a Search Strategy and Searching for Evidence. AJN The American Journal of Nursing. 2014;114(5).
36. Thorn H, Uhrenfeldt L. Experiences of non-specialist nurses caring for patients and their significant others undergoing transitions during palliative end-of-life cancer care: a systematic review. JBI Database System Rev Implement Rep. 2017;15(6):1711-46.
37. Graetz DE, Garza M, Rodriguez-Galindo C, Mack JW. Pediatric cancer communication in low- and middle-income countries: A scoping review. Cancer. 2020;126(23):5030-9.
38. EBSCO Information Services. EBSCOhost Research Platform 2023 [Available from: https://www.ebsco.com/products/ebscohost-research-platform.
39. Clarivate. EndNote 20 2023 [
40. Veritas Health Innovation. Covidence systematic review software, Melbourne, Australia.2023 [Available from: Available at www.covidence.org.
41. Peterson J, Pearce PF, Ferguson LA, Langford CA. Understanding scoping reviews: Definition, purpose, and process. J Am Assoc Nurse Pract. 2017;29(1):12-6.
42. Pollock D, Davies EL, Peters MDJ, Tricco AC, Alexander L, McInerney P, et al. Undertaking a scoping review: A practical guide for nursing and midwifery students, clinicians, researchers, and academics. Journal of Advanced Nursing. 2021;77(4):2102-13.
43. Munn Z, Stone JC, Aromataris E, Klugar M, Sears K, Leonardi-Bee J, et al. Assessing the risk of bias of quantitative analytical studies: introducing the vision for critical appraisal within JBI systematic reviews. JBI Evidence Synthesis. 2023;21(3):467-71.

For peer review only

For peer review only - http://bmjopen.bmj.com/site/about/guidelines.xhtml

AUTHORS' CONTRIBUTION

CK conceptualised the study, developed the methods, developed the preliminary search strategy, wrote the first draft, critically reviewed the draft manuscript and approved the final draft for submission.

LH conceptualised the study, developed the methods, developed the preliminary search strategy, critically reviewed the draft manuscript and approved the final draft for submission.

JM conceptualised the study, critically reviewed the draft manuscript and approved the final draft for submission.

NC provided critical inputs to methods and editing of this protocol, critically reviewed the draft manuscript and approved the final draft for submission.

COMPETING INTERESTS

Non declared.

FUNDING

This research received no specific grant from any funding agency in the public, commercial or not-for-profit sector.

For peer review only - http://bmjopen.bmj.com/site/about/guidelines.xhtml

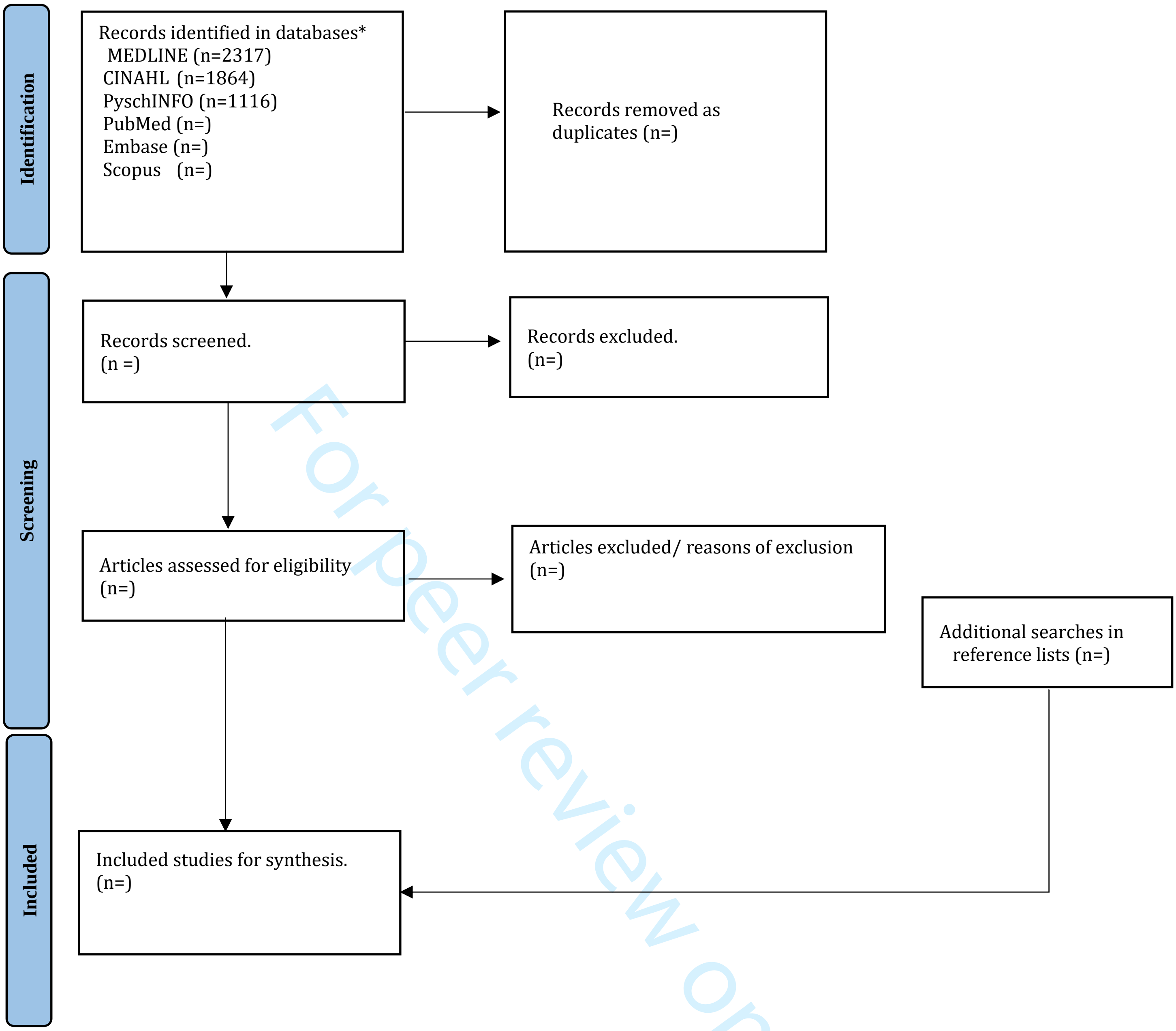

For peer review only

Appendix 1 Scoping search results

On 24 October 2022, a scoping search was conducted to check if this scoping review (or any review) is in process or has been conducted in the past. JBI systematic review register, Prospero, Cochrane register, and PubMed was searched using the simple search term "cancer communication". The table below summarises the findings:

Database	Summary
Prospero	The search retrieved nine records.<br>No registered review of the proposed topic.
JBI systematic review register	Only three articles were retrieved. No article included cancer communication.
PubMed	After filtering by meta-analysis, reviews, and systematic reviews, the search term "cancer communication*" yielded 136 studies. No review was similar to this scoping review objectives.
Cochrane	The search retrieved 35 records. No review was similar to this scoping review objectives.

For peer review only

Appendix 2 Initial data extraction

MEDLINE

1	Describing the population of this study	Results
Participants	(MH "Nursing Staff+") OR (MH "Nurses+") OR (MH "Nursing+") OR (MH "Nursing Care+") OR (MH "Nurse Practitioners+") OR (MH "Nursing Staff, Hospital+") OR "nursing staff" OR nurs* OR "nursing care" OR "nurse practitioner*" OR "hospital nurse*" OR "non-speciali#ed nurse*"	633,365
2	Describing the concepts –Cancers	
Cancers	(MH "Neoplasms+") OR neoplasm* OR neoplasia Or tum#r* OR "Malignant neoplasm*" OR Malignan* OR cancer* OR "cancer patient*"	4,718,229
3	Describing the concepts-communication and experiences	
Communication and communication experience	(MH "Communication+") OR (MH "Communication Methods, Total") OR (MH "Disclosure+") OR communication OR "information exchange" OR "communication pattern*" OR "communication strategy*" OR "communication method*" OR	3,060,614

For peer review only

	"communication technique*" OR disclosure OR interaction* OR experience*	
4	Combining all concepts	
	1 AND 2 AND 3	9,478
5	Limiter 1: Date between 2012 and June 2022	4,962
6	Limiter 2: adult 19 years and above	2,393
7	Limiter 3: English publication	2,317

CINAHL

1	Describing the population of this study	Results
Participants	(MH "Nurses+") OR (MH "Nursing Care+") OR “nursing staff” OR nurse* OR "nursing care" OR "nurse practitioner*" OR "hospital nurse*" OR "non-speciali#ed nurse*."	741,262
2	Describing the concepts –Cancers	
Cancers	(MH "Neoplasms+") OR (MH "Cancer Patients") OR neoplasm* OR neoplasia Or tum#r* OR “Malignant neoplasm*” OR	795,776

For peer review only

	Malignan* OR cancer* OR "cancer patients"	
3	Describing the concepts-communication and experiences	
Communication and communication experience	(MH "Communication+") OR (MH "Communication Methods, Total") OR (MH "Disclosure+") OR communication OR "information exchange" OR "communication pattern*" OR "communication strategy*" OR "communication method*" OR "communication technique*" OR disclosure OR interaction* OR experience*	895,801
4	Combining all concepts	
	1 AND 2 AND 3	9,442
5	Limiter 1: Date between 2012 and June 2022	5,188
6	Limiter 2: all adults (18 and above)	1,940
7	Limiter 3: English publication	1,864

For peer review only

PsycINFO

1	Describing the population of this study	Results
Participants	MM "Nursing" OR DE "Nurses" OR DE "Psychiatric Nurses" OR DE "Public Health Service Nurses" OR DE "School Nurses" OR "nursing staff" OR nurs* OR "nursing care" OR "nurse practitioner*" OR "hospital nurse*" OR "non-speciali#ed nurse*"	111,885
2	Describing the concepts –Cancers	
Cancers	DE "Neoplasms" OR DE "Benign Neoplasms" OR DE "Breast Neoplasms" OR DE "Endocrine Neoplasms" OR DE "Leukemias" OR DE "Melanoma" OR DE "Metastasis" OR DE "Nervous System Neoplasms" OR DE "Terminal Cancer" OR neoplasia OR tum#r* OR "Malignant neoplasm*" OR Malignan* OR cancer* OR cancer patient*	90,760

For peer review only

3	Describing the concepts-communication and experiences	
Communication and communication experience	DE "Communication" OR DE "Augmentative Communication" OR DE "Communication Barriers" OR DE "Electronic Communication" OR DE "Interpersonal Communication" OR DE "Messages" OR DE "Nonverbal Communication" OR DE "Persuasive Communication" OR DE "Privileged Communication" OR DE "Scientific Communication" OR DE "Social Communication" OR DE "Verbal Communication" OR communication OR “information exchange” OR “communication pattern*” OR “communication strategy*" OR "communication method*" OR "communication technique*" OR disclosure OR interaction* OR experience*	1,183,559
4	Combining all concepts	
	1 AND 2 AND 3	2,450

For peer review only

5	Limiter 1: Date between 2012 and June 2022	1,413
6	Limiter 2: adult 18 years and above	1,125
7	Limiter 3: English publication	1,116

For peer review only

PRISMA-P 2015 Checklist

This checklist has been adapted for use with protocol submissions to *Systematic Reviews* from Table 3 in Moher D et al: Preferred reporting items for systematic review and meta-analysis protocols (PRISMA-P) 2015 statement. *Systematic Reviews* 2015 **4**:1

Section/topic	#	Checklist item	Information reported		Line number(s)
			Yes	No	
**ADMINISTRATIVE INFORMATION**					
**Title**					
Identification	1a	Identify the report as a protocol of a systematic review	☒	☐	Page 1, line 1
Update	1b	If the protocol is for an update of a previous systematic review, identify as such	☐	☐	NA
**Registration**	2	If registered, provide the name of the registry (e.g., PROSPERO) and registration number in the Abstract	☒	☐	Page 3, line 3
**Authors**					
Contact	3a	Provide name, institutional affiliation, and e-mail address of all protocol authors; provide physical mailing address of corresponding author	☒	☐	Page 1, line 6-10
Contributions	3b	Describe contributions of protocol authors and identify the guarantor of the review	☒	☐	Page 21, line 1-12
**Amendments**	4	If the protocol represents an amendment of a previously completed or published protocol, identify as such and list changes; otherwise, state plan for documenting important protocol amendments	☐	☐	NA
**Support**					
Sources	5a	Indicate sources of financial or other support for the review	☒	☐	Page 21, line 17-20
Sponsor	5b	Provide name for the review funder and/or sponsor	☐	☐	NA
Role of sponsor/funder	5c	Describe roles of funder(s), sponsor(s), and/or institution(s), if any, in developing the protocol	☐	☐	NA
**INTRODUCTION**					
**Rationale**	6	Describe the rationale for the review in the context of what is already known	☒	☐	Page 4,5 and 6
**Objectives**	7	Provide an explicit statement of the question(s) the review will address with reference to participants, interventions, comparators, and outcomes (PICO)	☒	☐	Page 7 line 4-20

For peer review only

BioMed Central
The Open Access Publisher

Section/topic	#	Checklist item	Information reported		Line number(s)
			Yes	No	
**METHODS**					
**Eligibility criteria**	8	Specify the study characteristics (e.g., PICO, study design, setting, time frame) and report characteristics (e.g., years considered, language, publication status) to be used as criteria for eligibility for the review	☒	☐	Page 8, line 9-17 and Table 1
**Information sources**	9	Describe all intended information sources (e.g., electronic databases, contact with study authors, trial registers, or other grey literature sources) with planned dates of coverage	☒	☐	Page 10, line 3 to page 11, line 16
**Search strategy**	10	Present draft of search strategy to be used for at least one electronic database, including planned limits, such that it could be repeated	☒	☐	See Table 2 on page 12
STUDY RECORDS					
Data management	11a	Describe the mechanism(s) that will be used to manage records and data throughout the review	☒	☐	Page 14, line 1 to page 15, line 12.
Selection process	11b	State the process that will be used for selecting studies (e.g., two independent reviewers) through each phase of the review (i.e., screening, eligibility, and inclusion in meta-analysis)	☒	☐	Page 13, Line 7-21
Data collection process	11c	Describe planned method of extracting data from reports (e.g., piloting forms, done independently, in duplicate), any processes for obtaining and confirming data from investigators	☒	☐	Page 15 line 13 to Page 16 line 6
**Data items**	12	List and define all variables for which data will be sought (e.g., PICO items, funding sources), any pre-planned data assumptions and simplifications	☒	☐	Page 14, line 1 to page 15, line 12.
**Outcomes and prioritization**	13	List and define all outcomes for which data will be sought, including prioritization of main and additional outcomes, with rationale	☒	☐	Page 14, line 1 to page 15, line 12.
**Risk of bias in individual studies**	14	Describe anticipated methods for assessing risk of bias of individual studies, including whether this will be done at the outcome or study level, or both; state how this information will be used in data synthesis	☒	☐	Page 15, line 4 to 12.
DATA					
**Synthesis**	15a	Describe criteria under which study data will be quantitatively synthesized	☐	☐	NA

BioMed Central The Open Access Publisher

For peer review only

Section/topic	#	Checklist item	Information reported Yes	No	Line number(s)
	15b	If data are appropriate for quantitative synthesis, describe planned summary measures, methods of handling data, and methods of combining data from studies, including any planned exploration of consistency (e.g., I^2, Kendall's tau)	☐	☐	NA
	15c	Describe any proposed additional analyses (e.g., sensitivity or subgroup analyses, meta-regression)	☐	☐	NA
	15d	If quantitative synthesis is not appropriate, describe the type of summary planned	☒	☐	Page 15 line 14-18
**Meta-bias(es)**	16	Specify any planned assessment of meta-bias(es) (e.g., publication bias across studies, selective reporting within studies)	☐	☐	NA
**Confidence in cumulative evidence**	17	Describe how the strength of the body of evidence will be assessed (e.g., GRADE)	☐	☐	NA

NB: NA Not applicable

For peer review only

BioMed Central The Open Access Publisher
